# BCR::ABL1‐Induced Enhancer Reprogramming Uncovers Hypersensitivity of Ph+B‐ALL Cells to Enhancer‐Targeting Drugs

**DOI:** 10.1002/advs.202517231

**Published:** 2026-03-01

**Authors:** Han Leng Ng, Trudy Lee Glaser, Jintao Zhu, Mark E. Robinson, Kadriye N. Cosgun, Valeriya Malysheva, Ozgen Deniz, Nicholas T. Crump, Kaiyue Helian, Andrew J. Innes, Richard Burt, Li Sun, George John, Haibin Zhou, Atsunori Kaneshige, Longchuan Bai, Shaomeng Wang, Mikhail Spivakov, Markus Müschen, Niklas Feldhahn

**Affiliations:** ^1^ Centre for Haematology Department of Immunology and Inflammation Faculty of Medicine Imperial College London London UK; ^2^ Center of Molecular and Cellular Oncology Yale University New Haven Connecticut USA; ^3^ MRC London Institute of Medical Sciences London UK; ^4^ Institute of Clinical Sciences Faculty of Medicine Imperial College London UK; ^5^ VIB Center for Molecular Neurology, University of Antwerp, Belgium VIB Center for AI and Computational Biology Leuven Belgium; ^6^ Barts Cancer Institute Queen Mary University of London London UK; ^7^ The Hugh and Josseline Langmuir Centre for Myeloma Research Imperial College London London UK; ^8^ Department of Internal Medicine Medical School University of Michigan Ann Arbor Michigan USA

**Keywords:** BCR::ABL1, CBP/P300 inhibitors, enhancer reprogramming, leukemia, Ph+B‐ALL

## Abstract

Cancer is driven by genomic lesions and malignancy‐promoting transcriptional programs. In blood cancers, both are often interconnected as lesions frequently affect transcription factor (TF)‐encoding genes. TFs largely function through enhancers, and enhancer deregulation is linked to cancer initiation and progression. Consequently, enhancer‐targeting drugs are in trials for several advanced hematologic cancers. However, for cancers not driven by TF‐related lesions, it is less clear how their transcriptional programs are established; if oncogenesis involves enhancer‐deregulation, and if they are sensitive to therapeutic enhancer‐targeting. Here, we explore this for Philadelphia chromosome‐positive (Ph+) B‐lineage leukemia (B‐ALL), the most common B‐ALL in adults with a historically poor prognosis. Ph+B‐ALL is driven by BCR::ABL1, a kinase without TF‐related function. We report that malignant transformation and transcriptional reprogramming by BCR::ABL1 is indeed defined by enhancer reprogramming and that enhancer signatures differentiate Ph+B‐ALL from other leukemias. Mechanistically, we show that BCR::ABL1 itself induces enhancer activation, through its kinase activity and via kinase‐dependent activation of STAT5, ETV5, and MYC. Consequently, BCR::ABL1‐induced genes are hypersensitive to enhancer inhibition, and Ph+B‐ALL cells are hypersensitive to enhancer‐targeting drugs. Enhancer‐targeting further improves the efficacy of BCR::ABL1 kinase inhibitors used for Ph+B‐ALL therapy, especially in cells from *IKZF1* ^PLUS^ patients that most frequently relapse from current treatment, suggesting enhancer‐targeting as a potential promising addition to current therapy.

## Graphical abstract

Ng et al. show that the BCR::ABL1 kinase that drives the lymphoid leukemia Ph+B‐ALL modulates enhancer function by coopting signaling‐inducible transcription factors such as MYC, STAT5, and ETV5. BCR::ABL1 thereby promotes the transcriptional program driving and defining this leukemia and renders Ph+B‐ALL cells hypersensitive to enhancer‐inhibiting drugs currently tested in clinical trials.

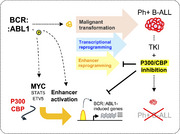

## 1 Introduction

Acute‐lymphoblastic leukemia (ALL) is the most common cancer, the second most common cause of cancer‐related death in children, and mostly arises from B‐cell precursors. B‐ALL subtypes are defined and driven by distinct initiating genetic lesions and disease‐defining transcriptional programs [1, 2]. Both driving factors are often interconnected, as most initiating genetic lesions in B‐ALL deregulate transcription factor (TF)‐encoding genes, which thereby cause the disease‐defining transcriptional program (13/18 [∼72%] B‐ALL subtypes with defined genetic alterations, excluding iAMP21 and hyper/hypodiploidy, are caused by lesions that alter one or two TFs [2]). TFs largely mediate their function through enhancers to allow temporal‐, spatial‐, and cell‐type‐specific gene activation. As enhancer function decreases with increasing distance to the promoter [3], promoters and enhancers maintain proximity through DNA looping, though examples exist where looping could not be detected [4, 5]. Importantly, cancer initiation and progression are frequently attributed to aberrant enhancer function such as enhancer reprogramming [6, 7, 8], enhancer hijacking [9, 10], or *de novo* enhancers [11, 12]. Given the importance of enhancer deregulation in cancer, enhancer‐targeting drugs have been explored over the last two decades as a therapeutic strategy for cancer treatment, with improved drug candidates being currently in clinical trials for advanced and high‐risk cancers, including hematological malignancies such as multiple myeloma (MM) and acute myeloid leukemia (AML) [13, 14].

Philadelphia chromosome‐positive B‐ALL (Ph+B‐ALL) is the most common B‐ALL in adults and historically a poor prognosis/high‐risk leukemia [15]. Recent advances in replacing, supplementing, or consolidating chemotherapy with tyrosine kinase inhibitors (TKIs) and immunotherapy have largely improved its treatment outcomes [16]. Nevertheless, relapses remain frequent and are associated with poor prognosis, particularly affecting *IKZF1* ^PLUS^ patients [16], where leukemia cells harbor secondary *IKZF1* deletions co‐occurring with deletions in *CDKN2A*, *CDKN2B*, *PAX5*, or *PAR1* in the absence of *ERG* deletion [17]. The primary driving lesion of Ph+B‐ALL is the oncogenic fusion gene *BCR::ABL1*, encoding a constitutively active tyrosine kinase that activates many signaling pathways important for survival and proliferation of Ph+B‐ALL cells. BCR::ABL1 expression also causes deregulation of the transcriptional program in transformed cells, leading to upregulation of genes essential for disease initiation and progression [18, 19, 20, 21, 22, 23, 24, 25, 26, 27]. However, how BCR::ABL1 induces this disease‐defining transcriptional program is poorly understood. Likewise, it is unknown if BCR::ABL1 deregulates enhancer function and if Ph+B‐ALL cells are consequently sensitive to enhancer‐targeting drugs.

While BCR::ABL1 has no TF‐related function, several TFs were shown important for Ph+B‐ALL cells. For example, BCR::ABL1 phosphorylation activates STAT1/3/5/6 [28, 29, 30], which is then thought to induce *MYC* [18, 19, 20]. Additionally, BCR::ABL1 induces RAS/MEK/ERK signaling, thereby inducing ETV5 [21, 31], JUN/FOS [32], and XBP1 [22]. Furthermore, MYB [33], CDK6 [33], CDK8 [34], FOXM1 [35], and ERG [36] were shown to be important for B‐ALL, including Ph+B‐ALL. However, it is unclear if these TFs are induced by BCR::ABL1 and thereby shape the Ph+B‐ALL‐defining transcriptome, or if their importance reflects a general requirement in B‐ALL cells. Likewise, for all Ph+B‐ALL‐linked TFs, it is generally unclear what their exact contribution to the Ph+B‐ALL‐defining transcriptional program is, and if they deregulate enhancer function to mediate their effect on Ph+B‐ALL cells.

To better understand how BCR::ABL1 establishes the Ph+B‐ALL‐defining transcriptional program, we performed an integrative multi‐omics approach to study the transcriptome, enhancer activities, and 3D interactions of enhancers with target genes in Ph+B‐ALL cells. We performed an in‐depth analysis of cells from human Ph+B‐ALL patients and a murine Ph+B‐ALL model using ChIP‐Seq, RNA‐Seq, and Hi‐C‐based methods to link enhancers to the promoters they regulate. We further interfered with enhancer function to explore their role and investigated BCR::ABL1‐regulated TFs that are recruited to them using targeted proteasomal degradation and RNA interference (RNAi).

## 2 Results

### 2.1 BCR::ABL1‐Induced Malignant Transformation and Transcriptional Reprogramming Is Associated with Genome‐Wide Enhancer Activation

**FIGURE 1:**
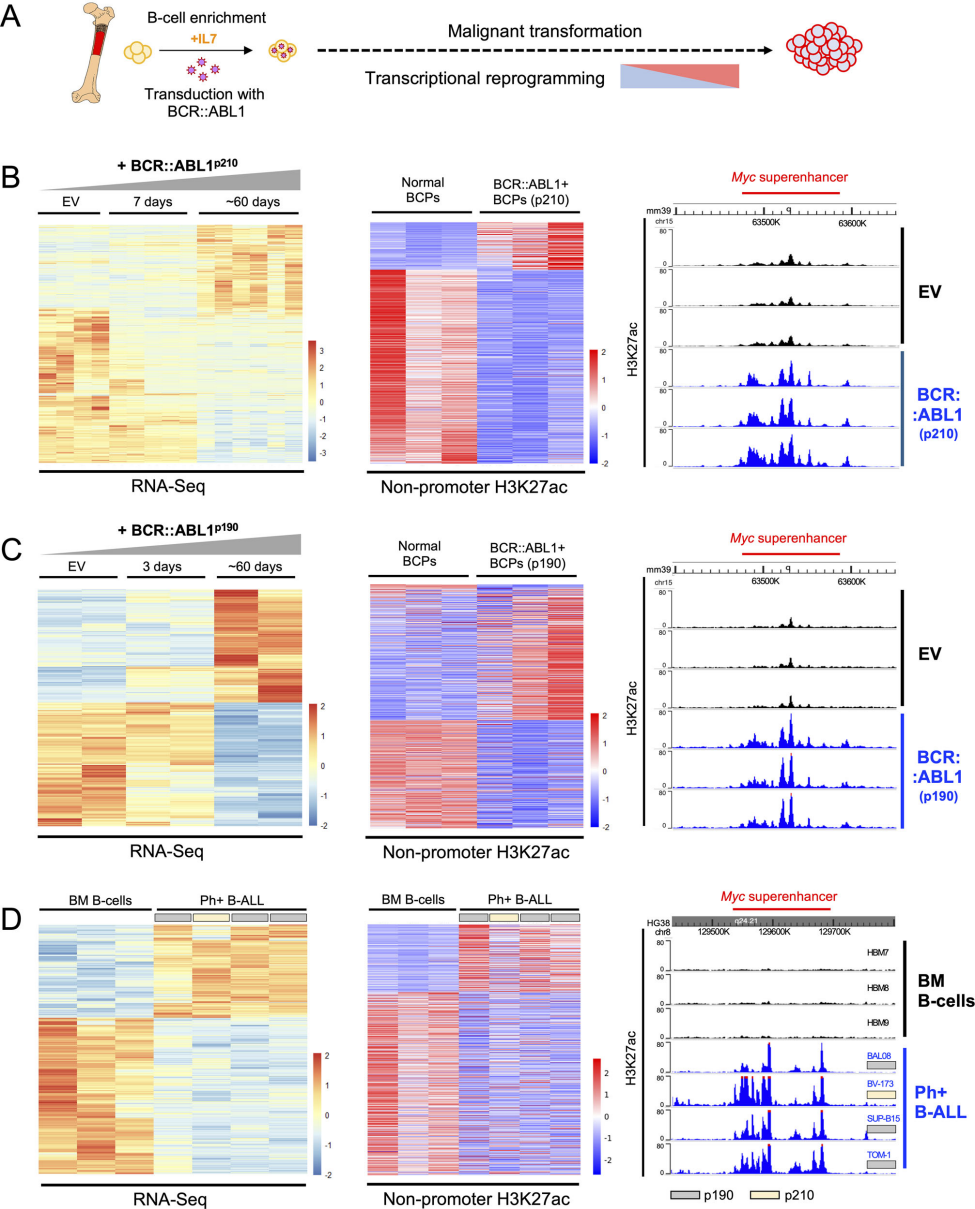
BCR::ABL1‐induced malignant transformation is associated with H3K27ac‐defined changes in enhancer activation. (A) A schematic of the experiments performed in this figure is shown. (B) (Left) A heatmap is shown visualizing differentially expressed genes (DEGs identified with RNA‐Seq) during malignant transformation of B‐cell precursors by BCR::ABL1. B‐cell precursors (BCPs) isolated from *Trp53bp1* ^−/−^ mice for this experiment were transduced with either MIGR1 empty vectors (EV) or BCR::ABL1 (p210) vectors and analyzed 7 days post‐transduction. BCR::ABL1 transduced cells were additionally analyzed at ∼60 days. (Middle) Normal BCPs (EV) and fully transformed leukemic BCPs (∼60d BCR::ABL1) were further assessed by H3K27ac ChIP‐Seq, and differential H3K27ac signals at non‐promoter locations are shown in this heatmap. (Right) Custom tracks of differential H3K27ac signals at the *Myc* super‐enhancer in BCR::ABL1 (p210) expressing cells are shown as an example. (C) (Left) A heatmap is shown visualizing differentially expressed genes (DEGs identified with RNA‐Seq) during malignant transformation of B‐cell precursors by BCR::ABL1 but using BCPs isolated from C57BL/6 mice and with BCR::ABL1 (p190). (Middle) Differential H3K27ac signals at non‐promoter locations for normal and fully transformed leukemic BCPs are shown. (Right) Custom tracks of differential H3K27ac signals at the *Myc* super‐enhancer in BCR::ABL1 (p190) expressing cells are shown as an example as in (B). (D) RNA‐Seq (left) and ChIP‐Seq (middle) heatmaps and H3K27ac ChIP‐Seq custom tracks (right) are shown as in (B/C) but for human Ph+B‐ALL cells in comparison to bone marrow (BM) B‐cells.

To investigate if BCR::ABL1 expression causes enhancer deregulation, we first monitored BCR::ABL1‐induced malignant transformation and associated transcriptional reprogramming for changes in enhancer activation using a murine transformation model based on viral transduction of ex vivo cultured primary B‐cell precursors (BCPs) with *BCR::ABL1*‐encoding retrovirus [21, 37, 38, 39]. We first re‐assessed RNA‐Seq and H3K27ac ChIP‐Seq data that we generated for a previous study [37] (Figure S1A,B). In this study, BCPs from *Trp53bp1* ^−/−^ mice were transduced with BCR::ABL1^p210^ (Figure 1A). Leukemic transformation was accompanied by substantial transcriptional reprogramming visualized by RNA‐Seq (Figure 1B, left; Table S1). BCR::ABL1‐induced genes included the known targets *Xbp1*, *Bcl2*, *Etv5*, *Dusp6*, and *Ccnd2*, while downregulated genes included many with B‐cell specific functions as previously described [40]. Changes in gene expression were accompanied by changes in enhancer activity as defined by non‐promoter H3K27ac signals (Figure 1B, middle), including increased signals at the *Myc* super‐enhancer in BCR::ABL1 expressing cells (Figure 1B, right). We repeated our setup using BCPs from C57BL/6 wild‐type mice and BCR::ABL1^p190^‐encoding retrovirus (Figure S1C,D and Table S2). This again showed that enhancer‐deregulation occurs during BCR::ABL1‐mediated transformation (Figure 1C). On average, BCR::ABL1 expression caused deregulation of ∼1,450 genes and 10,600 non‐promoter H3K27ac regions in B‐cell precursors from wild‐type mice. In line with this, human Ph+B‐ALL cells also showed defined changes in transcription and non‐promoter H3K27ac signals when compared to healthy bone marrow B‐cells (HBMs; Figure 1D).

### 2.2 Genes with Critical Functions in Ph+B‐ALL Are Enhancer‐Associated and Sensitive to Enhancer Targeting Drugs

**FIGURE 2:**
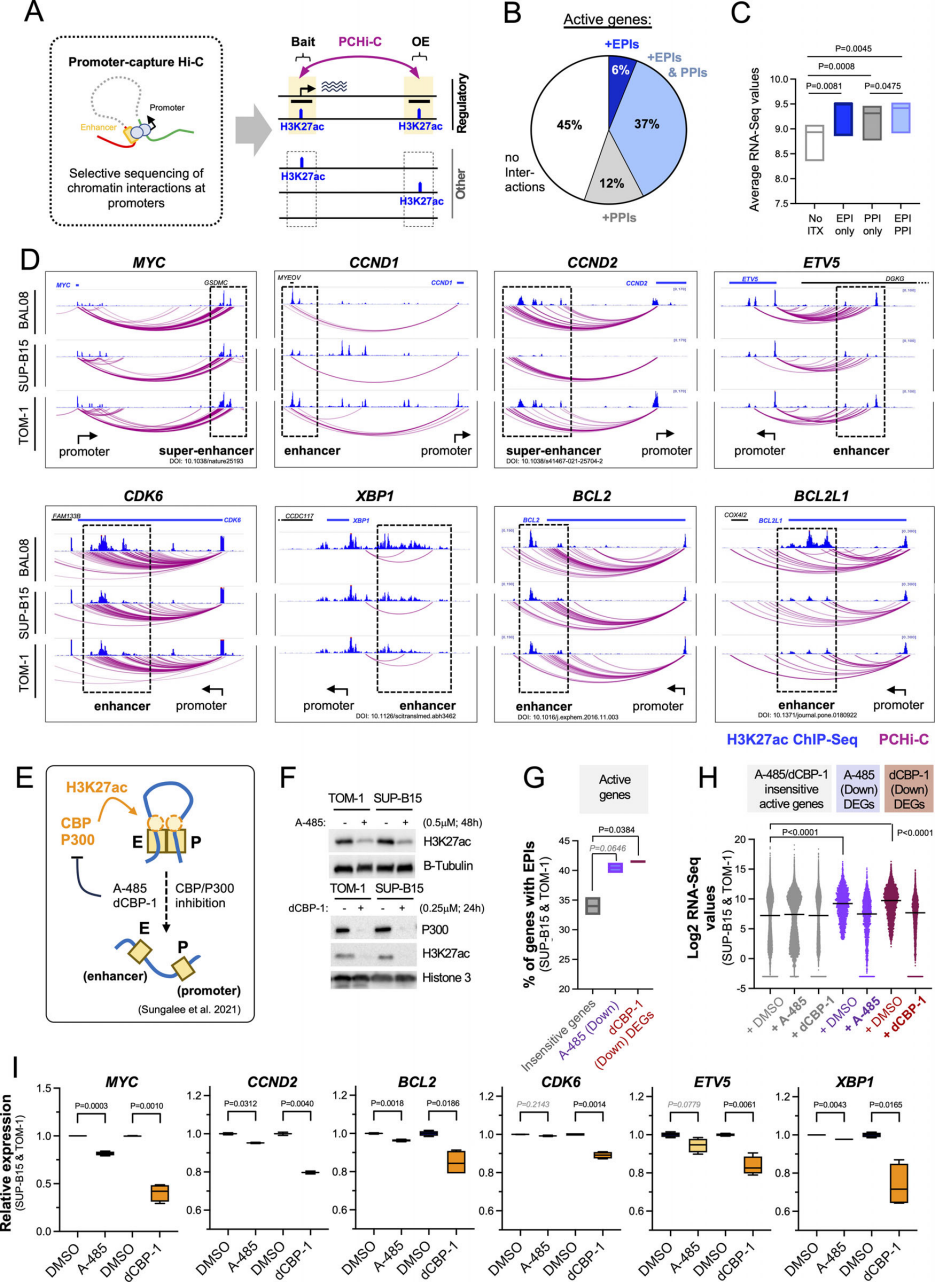
Key genes for Ph+B‐ALL cells are connected to enhancers through long‐range chromatin interactions. (A) Schematic of the analysis of enhancer‐promoter interactions in Ph+B‐ALL cells by Promoter‐Capture Hi‐C (PCHi‐C) and H3K27ac ChIP‐Seq is shown. The window on the right depicts potential chromatin interactions detected by this approach of active (H3K27ac+) promoters with active (H3K27ac+) other ends (OEs). (B) A pie chart is shown indicating the average percentage of active genes in Ph+B‐ALL cells (TOM‐1, SUP‐B15 and BAL08) that interact only with H3K27ac+ non‐promoter OEs (Enhancer‐Promoter interactions, EPIs, dark blue), only with other H3K27ac+ promoters (Promoter‐Promoter Interactions, PPIs only, grey), with EPIs and PPIs (EPIs&PPIs, light blue), or do not show interactions to H3K27ac+ OEs (white). (C) Bar chart showing average expression values of active genes with PCHi‐C interactions as described for (B) depicting average values from TOM‐1, SUP‐B15 and BAL08 cells. (D) Arc plots and custom tracks visualizing PCHi‐C‐defined chromatin interactions (purple lines) and H3K27ac signals (blue peaks) at active enhancers and promoters for selected genes with known key functions in Ph+B‐ALL cells are shown. Custom tracks were generated using the WashU epigenome browser and only interactions that start and end in the depicted area are shown. (E) A schematic is shown summarizing work from Sungalee et al. [47] that demonstrated that CBP/P300 inhibition interferes with enhancer function by reducing their physical interaction with promoters. (F) A Western blot is shown confirming the inhibitory function of A‐485 on CBP/P300 by visualizing H3K27ac levels (top) and the depletion of P300 and subsequent loss of H3K27ac by dCBP‐1 (bottom). (G/I) Resulting changes in gene expression were monitored in TOM‐1 and SUP‐B15 cells by RNA‐Seq and compared to promoter‐enhancer interactions defined by PCHi‐C. (G) Percentages of genes with EPIs are shown for genes that are sensitive to A‐485 or dCBP‐1 compared to genes insensitive to A‐485 or dCBP‐1. Data represents merged values from TOM‐1 and SUP‐B15 from n = 2 experiments each. (H) Average gene expression values are shown for genes sensitive/downregulated to A‐485 (purple) or dCBP‐1 (maroon) treatment compared to genes insensitive to A‐485 and dCBP‐1. Expression levels of DMSO‐treated, A‐485‐treated and dCBP‐1‐treated genes are shown alongside. Data represents merged values from TOM‐1 and SUP‐B15 from n = 2 experiments. (I) Relative expression values of DMSO, A‐485 or dCBP‐1‐treated cells are shown for six genes with crucial function in Ph+B‐ALL. Data represents RNA‐Seq values from TOM‐1 and SUP‐B15. To allow joint comparison, values were normalized to average DMSO. Statistical analysis in G‐I was performed using unpaired Student's *t*‐test and GraphPad PRISM.

To define which genes are enhancer‐regulated in Ph+B‐ALL, we performed promoter‐capture Hi‐C (PCHi‐C) [41, 42], which profiles long‐range chromatin interactions of promoters with distal DNA regions such as enhancers, and integrated PCHi‐C results with H3K27ac ChIP‐Seq to specifically define interactions between active promoters and active enhancers (Figure 2A). PCHi‐C and H3K27ac ChIP‐Seq were performed on two Ph+B‐ALL cell lines (SUP‐B15 and TOM‐1) and ex vivo cultured leukemia cells of one Ph+B‐ALL patient (BAL08; Figure S2A–F and Tables S3–S8). Long‐range PCHi‐C interactions for SUP‐B15 cells were comparable to those obtained by another study [43] that similarly assessed SUP‐B15 cells, supporting the reproducibility of our PCHi‐C data (Figure S2G). In line with previous work [44, 45], most active promoters were connected via long‐range chromatin interactions to distally located active regions (H3K27ac+ other ends/OEs; Figure S2H), and active promoters with H3K27ac+ OE interactions displayed a much higher number of long‐range chromatin interactions compared to those that only interacted with H3K27ac‐negative OEs (Figure S2I). A fraction of active genes with H3K27ac+ OE interactions solely interacted with other active promoters (i.e., Promoter‐Promoter Interactions/PPIs), however, most displayed interactions with H3K27ac+ non‐promoter regions (i.e., potential Enhancer‐Promoter Interactions/EPIs), either alone or in combination with PPIs (Figure 2B; Figure S2J). Genes with EPIs were generally higher expressed than genes without regulatory interactions (Figure 2C), and gene ontology (GO) analysis suggested they may serve distinct functions, with EPI+ only genes being largely enriched for signaling (Figure S2K). Importantly, while only 43% of active genes displayed EPIs by PCHi‐C (Figure 2B), these included many genes known to be regulated by BCR::ABL1 and/or to have critical functions in Ph+B‐ALL (Figure 2D).

To validate and assess PCHi‐C‐defined EPIs, we next inhibited CBP/P300 [46] and monitored resulting changes in gene expression by RNA‐Seq. CBP/P300 are essential for H3K27ac deposition [46], enhancer activation [46], and interaction of enhancers with promoters [47] (Figure 2E), and their inhibition causes transcriptional downregulation of enhancer‐regulated genes [47]. To interfere with CBP/P300, we used the CBP/P300 inhibitor A‐485 [48] and the CBP/P300 degrader dCBP‐1 [49] (Figure 2E,F; Figure S2L and Tables S9–S12) at concentrations that did not affect the viability of the treated cells at the time of analysis (Figure S2M). A‐485 and dCBP‐1 treatment caused deregulation of ∼11% and 20% of all active genes, respectively, and, in agreement with interfering with transcriptional activation, A‐485 and dCBP‐1 predominantly caused gene downregulation (∼67% of DEGs). It also affected lineage/cell type‐specific genes as previously described [50] (Figure S2N), and degradation was more effective than its inhibition (∼3,000 vs ∼1,600 DEGs, respectively). Genes with PCHi‐C‐defined EPIs were indeed preferentially downregulated by A‐485/dCBP‐1 when compared to active genes without regulatory interactions, and likewise genes with EPIs and PPIs showed preferential downregulation when compared to genes with PPIs only (Figure S2O–Q). Conversely, A‐485/dCBP‐1 sensitive genes were enriched for EPIs (Figure 2G) and, in line with EPI+ genes showing increased expression, A‐485/dCBP‐1 sensitive genes had a higher expression compared to insensitive genes (Figure 2H). Likewise, in line with the model that enhancers elevate pre‐existing basal levels of transcription [51], CBP/P300 inhibition reduced the expression of A‐485/dCBP‐1 sensitive genes to the level of A‐485/dCBP‐1 insensitive genes but did not abrogate their expression entirely (Figure 2H). Most importantly, however, and in line with our PCHi‐C data, A‐485/dCBP‐1 sensitive genes included EPI+ genes with key functions in Ph+B‐ALL (Figure 2I).

### 2.3 Enhancer Activation and Associated Chromatin Interactions Define Ph+B‐ALL Identity

**FIGURE 3:**
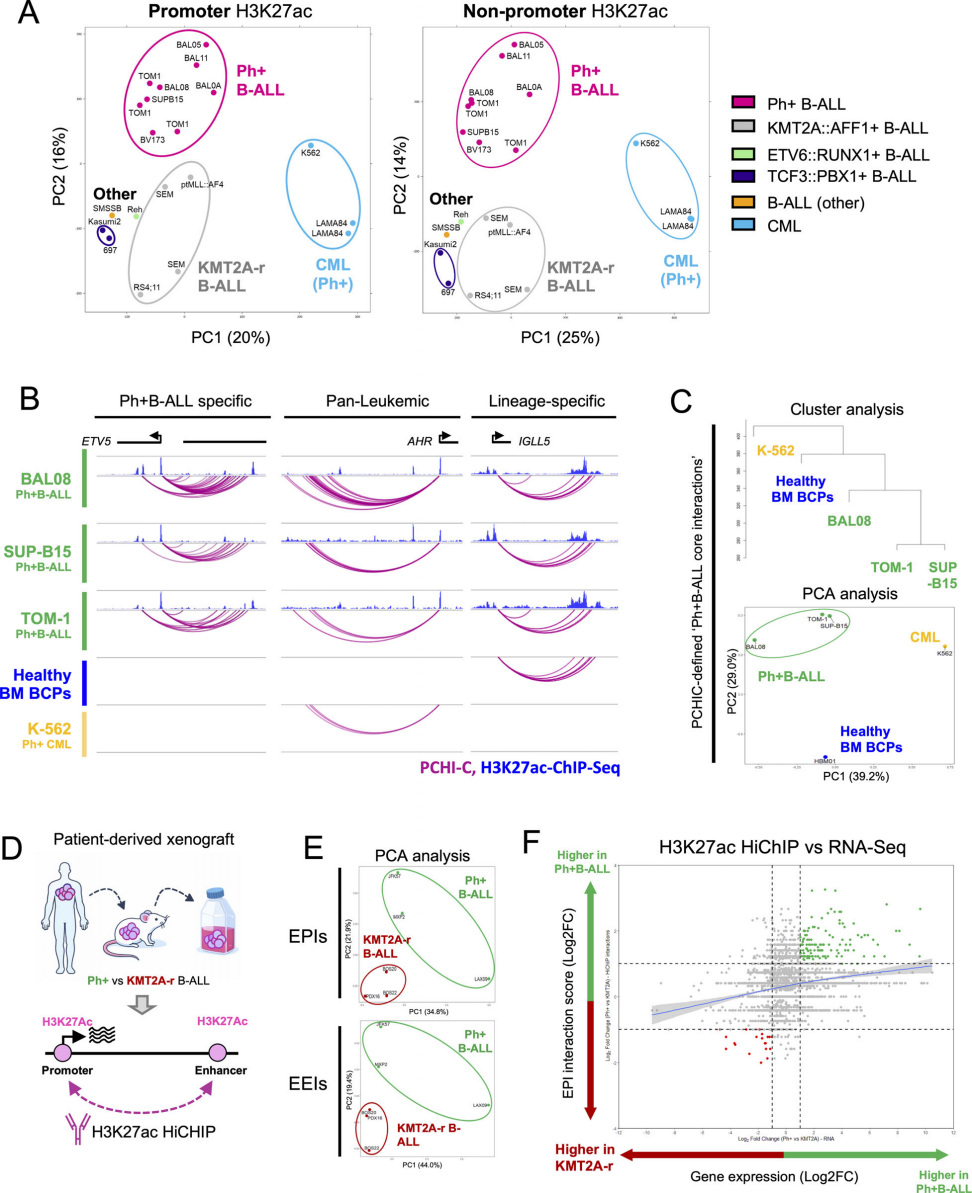
Enhancer‐promoter interactions define Ph+B‐ALL identity and mirror Ph+B‐ALL‐specific gene expression. (A) Principal component analysis (PCA) of H3K27ac ChIP‐Seq signals from cell lines and primary leukemia cells from patients for the indicated leukemia subtypes is shown. PCA analysis was performed using exclusively H3K27ac signals at promoters (left) or at non‐promoter regions (right). Leukemia subtypes of cell lines were defined using cytogenetic and phenotypic data from DSMZ. Data on *KMT2A::AFF1*+ B‐ALL cells, including ptMLL::AF4 were obtained from GSE74812, GSE71616 & GSE135024, while data on Ph+B‐ALL patients (i.e., BAL0A, BAL05, BAL08, BAL11) were generated by this study. (B) Arc plots of PCHi‐C interactions (purple lines) are shown for genes that show leukemia‐ and/or lineage‐specific PCHi‐C interactions. PCHi‐C data on three Ph+B‐ALL samples (BAL08 = patient, TOM‐1/SUP‐B15 = cell lines), and from healthy B‐cell precursors (BCPs) as well as Ph+ myeloid leukemia cells (K562) are shown. H3K27ac ChIP‐Seq custom (blue) tracks are added where available. Custom tracks were generated using the WashU epigenome browser and only interactions that start and end in the depicted area are shown. (C) A cluster dendrogram (top) and PCA plot (bottom) is shown using ‘Ph+B‐ALL CORE interactions’ that allow separation of Ph+B‐ALL cells from healthy BCPs and Ph+ myeloid leukemia cells. (D) A summary of the H3K27ac HiChIP experiment is shown. (E) PCA plots are shown for H3K27ac HiChIP defined enhancer‐promoter interactions (EPIs, top) and enhancer‐enhancer interactions (EEIs) for patient‐derived xenograft (PDX) Ph+ (n = 3) and *KMT2A::AFF1* (n = 3) B‐ALL cells. (F) A comparison of H3K27ac HiChIP defined EPIs and respective gene expression is shown, using log2FC values of EPIs per gene versus the expression of the respective genes for Ph+B‐ALL compared to *KMT2A::AFF1*+ B‐ALL. B‐ALL patient data from the TARGET study [1] was used for patient‐specific gene expression.

We next investigated if enhancer activation defines Ph+B‐ALL. Of note, B‐ALL subtypes can indeed be differentiated by ATAC‐Seq defined open chromatin [43], however, ATAC‐Seq peaks also include poised and repressed chromatin in addition to active enhancers. We therefore assessed enhancer activation here by specifically comparing non‐promoter H3K27ac signals or H3K27ac+ PCHi‐C interactions present in Ph+B‐ALL cells to those from other B‐ALLs and leukemia types. H3K27ac signals at non‐promoter regions clearly separated Ph+B‐ALL from Ph‐negative B‐ALLs and BCR::ABL1‐driven chronic myeloid leukemia (CML; Figure 3A). Next, we compared our PCHi‐C data from Ph+B‐ALL cells to PCHi‐C data that we generated for healthy CD19+CD10+ BM BCPs and CML cells (Figure 3B,C). Specifically, we defined Ph+B‐ALL‐specific H3K27ac+ ‘core interactions’ (i.e., EPIs and PPIs present in all three Ph+B‐ALL samples; Figure S3A and Table S13) and assessed if these chromatin interactions would discriminate Ph+B‐ALL cells from Ph‐negative cells. Again, both PCA and cluster analysis showed a distinct separation of Ph+B‐ALL cells from healthy BCPs and CML (Figure 3C). Notably, healthy BCPs clustered closer to Ph+B‐ALL samples than CML and closer to primary Ph+B‐ALL than to Ph+B‐ALL cell lines, reflecting their similarities in lineage type and cellular state. To challenge this, we assessed Ph+B‐ALL‐specific core interactions using a dataset on B‐ALL from Barnett et al. [43]. Also here, Ph+B‐ALL cells were separated from Ph‐negative cells by PCA analysis; notably, separation was better with H3K27ac‐defined core interactions compared to interactions solely overlapping with ATAC‐Seq peaks (Figure S3B). Lastly, to validate these observations with a different method and samples, we performed HiChIP using H3K27ac antibodies [52] on leukemia cells from three patient‐derived xenografts (PDX), each for Ph+B‐ALL and *KMT2A::AFF1*+ B‐ALL for comparison (Figure 3D–F). Like PCHi‐C, H3K27ac HiChIP visualizes EPIs but, additionally, also captures enhancer‐enhancer interactions (EEIs). In line with the above, H3K27ac HiChIP‐defined EPIs and EEIs efficiently separated Ph+B‐ALL from the *KMT2A::AFF1+* B‐ALL cells (Figure 3E). We further compared H3K27ac HiChIP‐derived EPI interaction scores per gene with RNA‐Seq expression of the respective genes. Specifically, we used log_2_ fold change (log2FC) values from the comparison of Ph+B‐ALL and *KMT2A::AFF1*+ B‐ALL cells to assess if their separation by EPIs and enhancer activation relates to respective differences in their gene expression programs. In line with enhancer activation defining cell‐type specific gene expression, this comparison showed a trend toward positive correlation of Ph+B‐ALL‐specific EPIs and the Ph+B‐ALL‐defining transcriptional program (Figure 3F).

### 2.4 BCR::ABL1 Induces Enhancer Activation through Its Kinase Activity

**FIGURE 4:**
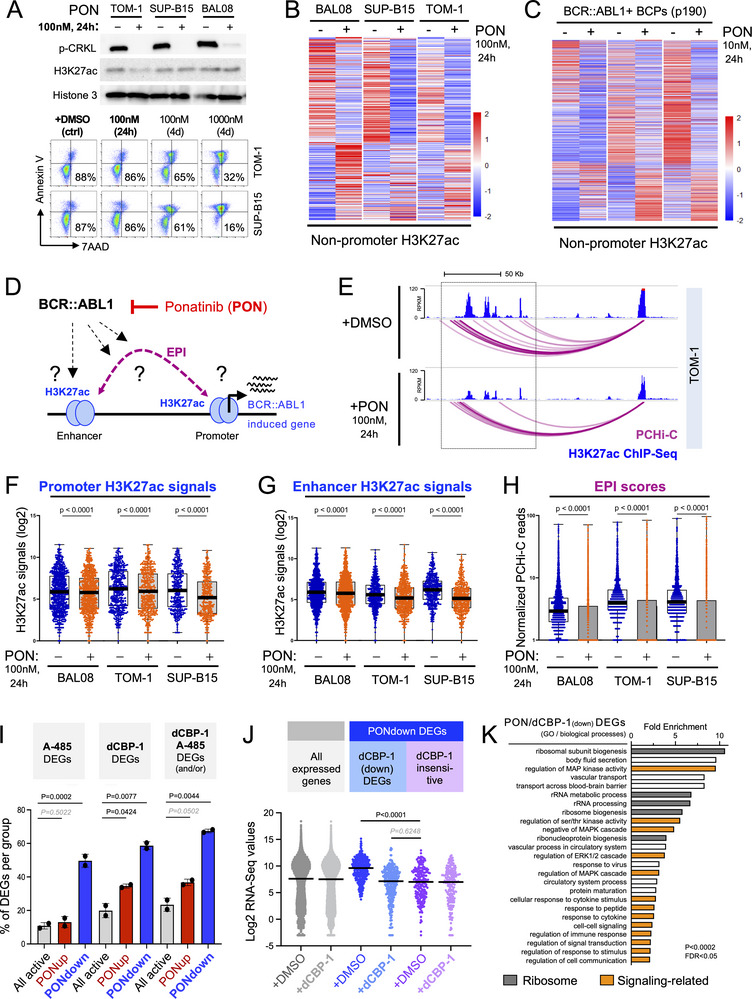
BCR::ABL1‐induced enhancer deregulation depends on BCR::ABL1 kinase activity, and BCR::ABL1‐induced genes are hypersensitive to enhancer‐targeting drugs. (A) (Top) A Western blot to validate the effectiveness of 24 h 100 nM Ponatinib (PON) treatment to inhibit BCR::ABL1 activity in Ph+B‐ALL cells is shown using the established BCR::ABL1 target CRKL as a positive control. (Bottom). Representative flow cytometry plots for the treatment are shown, with Annexin V and 7‐AAD indicating apoptotic and dead cells, respectively. As a positive control, cells treated with 100 nM or 1000 nM Ponatinib for 4 days are shown. (B/C) Heatmaps of H3K27ac ChIP‐Seq signals at non‐promoter regions are shown for DMSO‐ versus Ponatinib‐treated murine (B) and human (C) BCR::ABL1^p190^‐driven B‐ALL cells. Only regions with differential H3K27ac signals in all samples are plotted. (D) A schematic summarizing the analysis of EPIs and H3K27ac ChIP‐Seq signals at Ponatinib‐sensitive, downregulated genes with EPIs is performed for E‐H is shown. (E) Arc plots of PCHi‐C and H3K27ac ChIP‐Seq signals are shown for the known BCR::ABL1‐deregulated gene, *CCND2*. Data is shown for TOM‐1 cells treated with Ponatinib or DMSO as a control. (F/G) H3K27ac ChIP‐Seq signals at promoters (F) and enhancers (G) of Ponatinib‐sensitive, downregulated genes with EPIs are shown. (H) PCHi‐C scores of EPIs at Ponatinib‐sensitive, downregulated genes with EPIs are shown. Statistical analysis in F‐H was performed by paired Student's *t*‐test on GraphPad PRISM. Box‐and‐whisker plots for F‐H show the median (thick line), interquartile range (25^th^ – 75^th^ percentiles; box), and the minimum and maximum (whiskers). (I) Average percentages of A‐485 DEGs, dCBP‐1 DEGs, and A‐485/dCBP‐1 DEGs are shown for genes that are up‐ or downregulated by 24 h Ponatinib treatment, in comparison to all active genes. (J) Average gene expression values are shown for BCR::ABL1‐induced genes (i.e., Ponatinib‐downregulated / PONdown DEGs) that are sensitive or insensitive to dCBP‐1 in comparison to all active genes. Expression levels of DMSO‐treated and dCBP‐1‐treated genes are shown alongside. Data in I/J represents merged values from TOM‐1 and SUP‐B15 obtained from n = 2 independent RNA‐Seq experiments. Statistical analysis was performed using Student's *t*‐test and GraphPad PRISM. (K) The 25 most enriched Gene Ontology (GO) pathways (biological processes) for dCBP‐1 and Ponatinib‐downregulated genes are shown. Pathway associations to functional groups are indicated by color. Pathway names are shortened to match the figure width.

To investigate how Ph+B‐ALL cells enable enhancer activation, we next inhibited BCR::ABL1 kinase activity for 24 h using 100 nM Ponatinib and monitored its effect on enhancer activation through H3K27ac ChIP‐Seq. This treatment efficiently silences BCR::ABL1 (Figure 4A, top) but does not affect the viability of Ph+B‐ALL cells during the 24 h period (Figure 4A, bottom). However, Ponatinib caused substantial loss of H3K27ac signals at H3K27ac+ non‐promoter regions in human Ph+B‐ALL cells (Figure 4B) and BCR::ABL1^p190^‐transformed murine BCPs (Figure 4C), which was similarly observed when Asciminib was used to block BCR::ABL1 (Figure S4A). BCR::ABL1 kinase inhibition also reduced non‐promoter H3K27ac signals in BCR::ABL1‐driven myeloid leukemia cells (Figure S4B), indicating that the effect of BCR::ABL1 on enhancer regulation is not limited to Ph+B‐ALL. Ponatinib also increased H3K27ac at some enhancers, possibly reflecting resumed differentiation upon BCR::ABL1 inhibition as previously described [40, 53]. Next, we specifically analyzed genes that display PCHi‐C‐defined EPIs (Figure 4D,E), focusing on genes that are downregulated upon Ponatinib treatment by RNA‐Seq (i.e., Ponatinib‐downregulated / PONdown genes; Tables S14–S17). H3K27ac signals at promoters and enhancers were both significantly reduced upon Ponatinib treatment (Figure 4F,G), with its effect increasing with the proliferative state of the cells (i.e., BAL08 > TOM‐1 > SUP‐B15). Moreover, Ponatinib substantially reduced PCHi‐C‐defined promoter‐enhancer interactions at PONdown genes in all comparisons (Figure 4H).

### 2.5 BCR::ABL1‐Induced Genes Are Hypersensitive to Enhancer‐Targeting Drugs

Given that BCR::ABL1 regulates enhancer function at BCR::ABL1‐induced genes, we next wondered if BCR::ABL1‐induced genes are especially sensitive to enhancer inhibition by CBP/P300‐targeting drugs. Specifically, we assessed how many Ponatinib‐downregulated genes (i.e., BCR::ABL1‐induced) are likewise downregulated by A‐485 and/or dCBP‐1 using our RNA‐Seq data. Of note, A‐485 and dCBP‐1 largely affect the same genes but also showed individual sensitivities (Figure S2L). Remarkably, up to ∼70% of all BCR::ABL1‐induced genes were A‐485/dCBP‐1 sensitive, compared to only ∼20% of all active genes (Figure 4I). In line with our results from before, CBP/P300 inhibition‐sensitive PONdown genes were higher expressed than CBP/P300 inhibition‐insensitive PONdown genes, and CBP/P300 inhibition reduced their expression to levels of CBP/P300 inhibition‐insensitive genes (Figure 4J). In agreement with our gene ontology analysis of enhancer‐associated genes, CBP/P300 inhibition‐sensitive PONdown genes were further associated with signaling and translation (Figure 4K).

### 2.6 BCR::ABL1 Mediates Enhancer Activation through BCR::ABL1‐induced Action of STAT5, ETV5 and MYC

**FIGURE 5:**
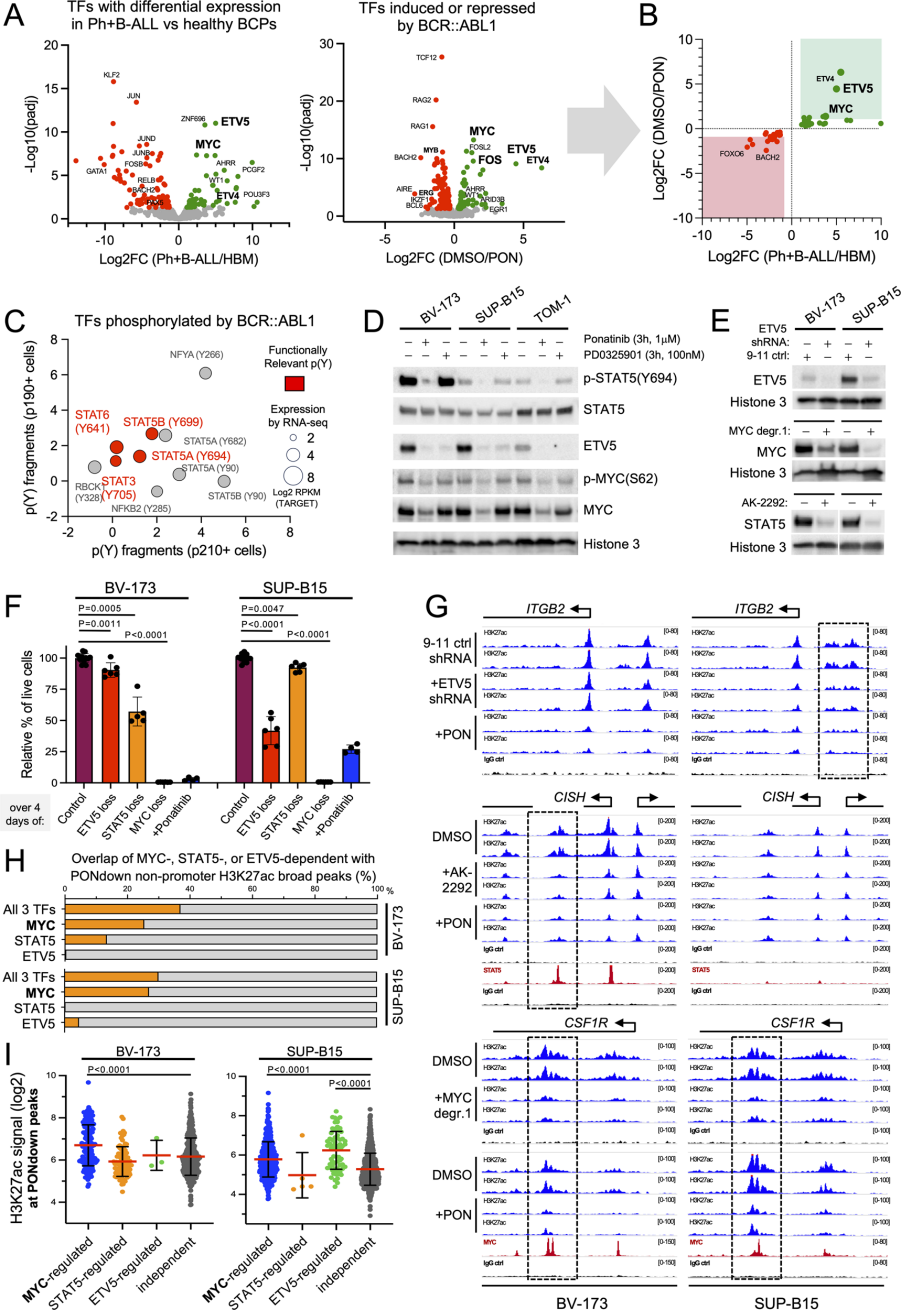
Enhancer activation through BCR::ABL1‐induced action of STAT5, ETV5, and MYC. (A‐C) Analysis of BCR::ABL1‐induced gene expression (A/B) or phosphorylation (C) for previously defined TF encoding genes [80]. (A) Volcano plots of RNA‐Seq data are shown, visualizing expression and related significance of TF‐encoding genes that are either up/down‐regulated in Ph+B‐ALL cells compared to healthy bone marrow B‐cell precursors [HBM] (left) or up/down‐regulated in DMSO‐treated compared to Ponatinib (PON) treated Ph+B‐ALL cells (right). For DMSO/PON, three Ph+B‐ALL cell lines (BV‐173, TOM‐1, SUP‐B15) and one Ph+B‐ALL patient (BAL08) were compared. For Ph+B‐ALL/HBM the same Ph+B‐ALL cells were compared to CD19+CD10+ HBMs isolated from n = 3 healthy donors. (B) Differential TFs induced or reduced in both plots of (A) are shown. (C) An XY plot visualizing BCR::ABL1‐phosphorylated TFs identified by two published mass spectrometry data sets [54, 55] is shown. X and Y axes indicate the p210/parental and p190/parental median normalized ratios from Cutler et al. [55] Dot sizes indicate the relative expression of the relative genes in Ph+B‐ALL cells using RNA‐Seq data from the TARGET study. Previously reported functionally relevant phospho‐tyrosines (00pY) are highlighted. Note that NFYA (Y266) is functionally irrelevant as described by Bernardini et al. [81]. (D) Representative Western blot images are shown for Ph+B‐ALL cell lines treated for 3 h with Ponatinib or the MEK inhibitor PD0325901 as indicated and using antibodies as indicated. Controls were DMSO‐treated throughout. Note, tyrosine‐694 phosphorylation of STAT5 indicates its induced activation, and serine‐62 phosphorylation of MYC its protein stabilization. (E) Western blot images are shown validating RNAi‐mediated suppression of ETV5, MYC degradation by A80.2HCl/MYC degrader 1, and AK‐2292‐mediated degradation of STAT5. For RNAi experiments, a C911 seed control was used, which is the same shRNA as the ETV5 targeting shRNA but mutated at nucleotide positions 9–11. C911 seed control shRNAs are predicted to lack on‐targeting activity but have the same off‐target activity [76]. Note that RNAi could not be applied to TOM‐1 cells due to their immediate, anti‐proliferative response to any viral transduction. (F) A bar diagram is shown visualizing relative numbers of live cells obtained from 4‐day Cell Titer Glo (CTG) experiments (n≥2 experiments with n = 2 technical replicates each). The diagram summarizes the effect of ETV5 shRNA, STAT5 degrader AK‐2292, MYC degrader 1, or Ponatinib treatment on BV‐173 and SUP‐B15 cells. Mean values are plotted +SD. Experiments are also shown as individual diagrams in Figure S5C. (G) ChIP‐Seq custom tracks are shown for H3K27ac and IgG as an antibody control. Additionally, ChIP‐Seq for STAT5 and MYC was performed with IgG as a control (ChIP‐Seq for ETV5 could not be performed due to the lack of a suitable antibody). N = 2 experiments are shown for 24 h treatment with 2.5 µM AK‐2292, 100 nM Ponatinib (PON), 25 nM MYC degrader 1 or DMSO (1:1000), or FACS‐sorted ETV5 shRNA and C9‐11 seed control shRNA expressing cells. Square boxes indicate reductions in non‐promoter H3K27ac. (H) A bar diagram is shown summarizing the percentage of DiffBind‐defined non‐promoter H3K27ac peaks significantly downregulated upon Ponatinib treatment (PONdown peaks) that overlap with non‐promoter H3K27ac peaks significantly downregulated upon MYC degrader 1 treatment (MYC), AK‐2292 treatment (STAT5), or ETV5‐shRNA expression (ETV5). (I) A bar diagram is shown visualizing H3K27ac peak signal intensities at PONdown non‐promoter H3K27ac peaks (in DMSO‐treated control cells) that do or do not overlap with MYC‐regulated, STAT5‐regulated, or ETV5‐regulated non‐promoter H3K27ac peaks as indicated. The red line represents the mean, with SD in black lines.

As BCR::ABL1 has no enhancer‐related function, we assessed which TFs are utilized by BCR::ABL1 to induce enhancer activation. We first investigated TFs previously linked to Ph+B‐ALL [18, 19, 20, 21, 31, 32, 33, 34, 35, 36, 39]. Specifically, we assessed them for being transcriptionally upregulated in Ph+B‐ALL and downregulated upon BCR::ABL1 kinase inhibition as characteristic of being BCR::ABL1‐induced. Surprisingly, only two of these (i.e., ETV5 and MYC) fulfilled this criterion (Figure S5A). Extending our analysis to all known TFs further identified ETV4 (Figure 5A,B), which, however, is expressed at much lower levels than ETV5 in Ph+B‐ALL cells (Figure S5B). Analysis of two mass spectrometry datasets [54, 55] for TFs activated by BCR::ABL1‐induced tyrosine phosphorylation additionally identified STAT3/5/6 (Figure 5C). Noteworthy, while not being direct phosphorylation targets of BCR::ABL1, ETV5 and MYC are also regulated by BCR::ABL1 kinase activity on the protein level, but through protein stabilization, as 3 h kinase inhibition substantially reduced their protein quantities (Figure 5D). As STATs, ETV5 and MYC were further previously linked to P300/CBP and/or enhancer activation [56, 57], we hypothesized these TFs as the most likely mediators of BCR::ABL1‐induced transcriptional activation and enhancer activation. To functionally explore them, we used targeted proteolytic degradation and RNA interference (RNAi) (Figure 5E). Regarding STAT3/5/6, we focused on STAT5 as murine models previously defined it as the main effector of BCR::ABL1 over other STAT family members [39, 58]. We first monitored the phenotypic effect of their loss on Ph+B‐ALL cells. Surprisingly, while STAT5 loss completely abrogates BCR::ABL1‐induced leukemia in mice [39, 58], its loss in human Ph+B‐ALL cells by AK‐2292‐induced degradation had relatively mild effects and varied between individual lines (Figure S5C); with additional depletion of STAT3 and STAT6 not further enhancing the effect of STAT5 loss (Figure S5D). Likewise, loss of ETV5 through RNAi had rather moderate effects and varied between individual lines (Figure S5C). Interestingly, cells that were sensitive to STAT5 loss were only mildly sensitive to ETV5 loss and vice versa (Figure 5F). While initially surprising, it fits with recent observations that dependencies on JAK/STAT and RAS/MEK/ERK pathways in B‐ALL are mutually exclusive [59] and that Ph+B‐ALL is comprised of three sub‐types (i.e., C1‐3) that are defined by differences in JAK/STAT, RAS/MEK/ERK and PI3K/AKT pathway activation [60] (ETV5 is induced by RAS/MEK/ERK). In line with this, ETV5 depletion‐sensitive SUP‐B15 cells showed a tendency toward the transcriptional program of the RAS/MEK/ERK‐defined C2 subgroup, while STAT5 depletion‐sensitive BV‐173 cells showed a tendency toward the JAK/STAT‐defined C1 subgroup (Figure S5E). In contrast to ETV5 and STAT5, MYC depletion had a detrimental effect on all Ph+B‐ALL lines tested (Figure 5F; Figure S5C).

Next, we explored the effect of their loss on BCR::ABL1‐induced enhancer activation. Loss of each TF reduced H3K27ac levels at BCR::ABL1‐induced regions, with ETV5 and STAT5 following the previously observed pattern of Ph+B‐ALL cell line subgroup specificity and MYC being required in both Ph+B‐ALL lines assessed (Figure 5G). MYC displayed the biggest contribution to BCR::ABL1‐induced enhancer regulation, with its loss affecting ∼25 and 27% of Ponatinib‐sensitive/PONdown H3K27ac regions in BV‐173 and SUP‐B15 cells, respectively (Figure 5H). In contrast, both STAT5 and ETV5 displayed Ph+B‐ALL‐subgroup specific enhancer regulation, with STAT5 regulating 13% of PONdown H3K27ac regions in BV173 (and 0% in SUP‐B15), and ETV5 regulating 5% of PONdown H3K27ac regions in SUP‐B15 (and 0% in BV173). Due to partial overlap of MYC with STAT5 or ETV5, the total contribution of all three TFs could be linked to ∼one‐third of all BCR::ABL1‐induced H3K27ac regions (Figure 5H), though their importance for enhancer regulation could be higher as especially MYC‐regulated BCR::ABL1‐induced H3K27ac regions displayed much higher H3K27ac signals than MYC/STAT5/ETV5‐unregulated PONdown regions (Figure 5I). Nonetheless, our results also show that most H3K27ac‐defined chromatin is regulated by BCR::ABL1 independent of MYC, STAT5, and ETV5.

### 2.7 Ph+B‐ALL Cells Are Hypersensitive to Therapeutic Enhancer‐Targeting

**FIGURE 6:**
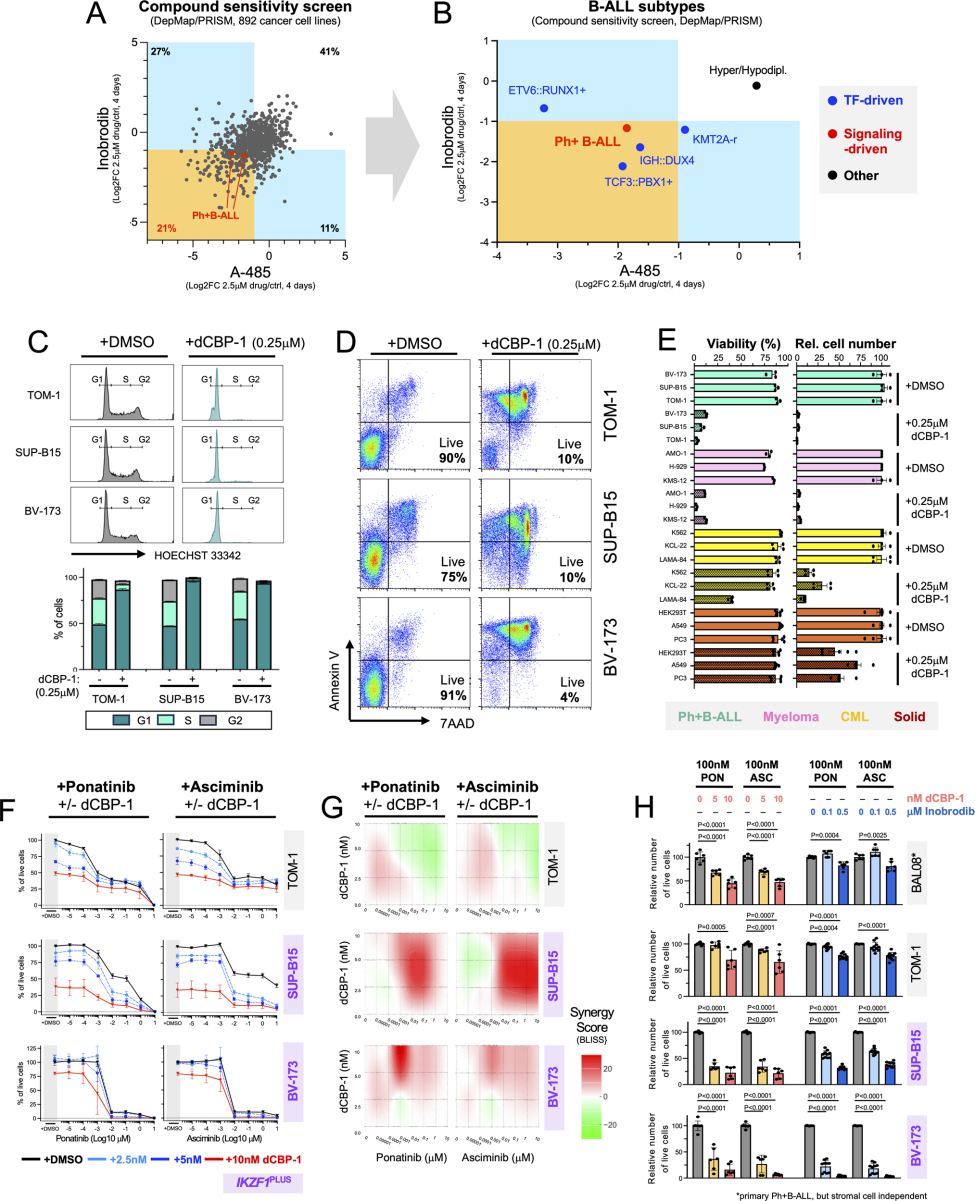
Ph+B‐ALL cells display hypersensitivity to enhancer targeting drugs. (A/B) XY plots of DepMap compound screen data are shown, indicating Log2FC of numbers of live cells for a 4‐day treatment with 2.5 µM Inobrodib/DMSO or A‐485/DMSO. Log2FC of <−1 was considered a sensitivity to the drug, highlighted in blue/orange. (A) Data on all 892 cancer cell lines from the compound screen is shown, with the two Ph+B‐ALL cell lines included in the screen indicated. (B) Average numbers for cell lines of the indicated B‐ALL subtypes are shown separately. (C) Cell‐cycle analysis of 48 h 0.25 µM dCBP‐1 treated Ph+B‐ALL cells (n = 2) by HOECHST 33342 staining is shown. (Top) Representative histograms are shown, indicating cells in G1, S, and G2 phases of the cell cycle. (Bottom) Bar diagram summarizing the results of two experiments. (D) Viability analysis of Ph+B‐ALL cells treated for five days with 0.25 µM dCBP‐1 using 7‐AAD/Annexin V staining is shown. Representative flow cytometry dot plots are shown with live cell percentages indicated (7‐AAD‐/Annexin V‐ cells, lower left quadrant). (E) A bar diagram is shown summarizing the results of 5‐day 0.25 µM dCBP‐1 treatments of Ph+B‐ALL cell lines (n = 3). Multiple myeloma, chronic myeloid leukemia (CML), and cancerous cell lines from solid tissues were treated and analyzed for comparison, with cell line names indicated in the figure. The mean and SD are plotted for each sample and treatment. (Left) Viability in % defined by 7‐AAD/Annexin V staining is shown. (Right) Relative cell numbers defined by cell counting/trypan blue exclusion are shown. (F) Dose response curves for Ponatinib and Asciminib on the indicated cell lines are shown. The percentage of live cells shown was defined by 4‐day treatment using the Cell Titer Glo (CTG) assay. Curves display single or combination treatments with dCBP‐1 as indicated. Data represents n≥3 experiments. (G) A synergy analysis for the combination treatment of Ponatinib or Asciminib with different concentrations of dCBP‐1 as indicated, is shown. XY plots show BLISS scores generated by *SynergyFinder* using the data from (F). LOEWE, HSA, and ZIP analyses can be found in Figure S6A. (H) Bar diagrams show the effect of low‐dose dCBP‐1 or Inobrodib addition to Ponatinib or Asciminib treatment of Ph+B‐ALL cells at Ponatinib/Asciminib concentrations equivalent to the standard dose given to Ph+B‐ALL patients. Specifically, the relative number of live cells determined by CTG after four days of drug treatment is shown, normalized to Ponatinib/Asciminib only treated cells. The mean value and SD are shown in the plot (n = 3–4 experiments and n = 2 technical repeats for each). Statistical analysis was performed using unpaired Student's *t*‐test and GraphPad PRISM.

Given that the contribution of TFs to enhancer activation in Ph+B‐ALL is rather complex, but most BCR::ABL1‐induced genes are sensitive to enhancer inhibition by CBP/P300‐targeting drugs, we next wondered if CBP/P300‐targeting could benefit Ph+B‐ALL patients. We first reassessed a public compound screen (DepMap [61]), in which 892 cancer cell lines were tested for sensitivities to established drugs, including the CBP/P300 inhibitors Inobrodib and A‐485. In agreement with enhancer function being crucial for cancer cells, 59% of cell lines in this screen showed sensitivity to these drugs. However, only ∼21% showed sensitivity to both drugs, notably including both Ph+B‐ALL cell lines tested in that screen (Figure 6A). Within B‐ALL, TF‐driven subtypes showed high sensitivity to CBP/P300 inhibitors, while TF‐unrelated hyper/hypodiploid B‐ALL did not (Figure 6B). Ph+B‐ALL exhibited sensitivity similar to TF‐driven subtypes. Thus, we investigated the effect of CBP/P300‐targeting on the viability and proliferation of Ph+B‐ALL cells in more depth. CBP/P300 targeting by dCBP‐1 caused near complete G1 cell cycle arrest within 2 days of treatment (Figure 6C) followed by subsequent apoptosis within 5 days (Figure 6D). In fact, Ph+B‐ALL cells displayed similar sensitivity to CBP/P300 targeting as multiple myeloma (MM) cells (Figure 6E), which were repeatedly described as the most CBP/P300‐inhibition sensitive cancer cells [48, 49, 62]. In line with the DepMap compound screen, the same treatment had little effect on the viability of cancerous cells from solid tissues tested here, and even BCR::ABL1‐driven myeloid leukemia (CML) cells showing little effect on viability (Figure 6E left). Though dCBP‐1 affected the proliferation of all cancerous cell lines tested, also here Ph+B‐ALL and MM cells also showed hypersensitivity compared to the other cell lines (Figure 6E right). In analogy to the successful addition of CBP/P300 targeting to standard treatment of multiple myeloma patients in a recent trial [63], we next assessed if CBP/P300 targeting could be used as combination therapy with TKIs for Ph+B‐ALL. CBP/P300 targeting through low‐dose dCBP‐1 treatment (2.5–10 nM) indeed enhanced the efficacy of the most recent TKIs, Ponatinib and Asciminib (Figure 6F), and showed synergy for two out of three Ph+B‐ALL lines studied here (Figure 6G; Figure S6A). Combined targeting was also effective when molar TKI ratios equivalent to plasma concentrations of standard dose treatments of Ph+B‐ALL patients were used (∼100 nM for Ponatinib/Asciminib) and using the clinically tested CBP/P300 Inhibitor Inobrodib (Figure 6H; Figure S6B). Furthermore, it appeared effective in Ph+B‐ALL cells from poor prognosis *IKZF1* ^PLUS^ patients (Figure 6H; Figure S6C–E) and for reportedly TKI‐insensitive cells such as SUP‐B15 [64, 65] (Figure 6F–H).

## 3 Discussion

Enhancer inhibition is explored in many cancers as a therapeutic strategy, with BET inhibitors being tested for the last two decades [14] and CBP/P300 inhibitors currently in phase 1/2 clinical trials [13]. Enhancer function has also been studied in hematological malignancies such as AML, T‐ALL, MM, and B‐ALL [43, 66, 67]. However, B‐ALL subtype‐specific dependencies on enhancer function have not been thoroughly investigated, particularly regarding those not driven by altered TFs, such as Ph+B‐ALL. Our study is the first to explore this in depth. We demonstrate that enhancer regulation is a defining feature of Ph+B‐ALL and that Ph+B‐ALL cells are highly sensitive to enhancer‐targeting drugs. Enhancer deregulation is part of the process of malignant transformation and is executed by BCR::ABL1 itself for BCR::ABL1‐regulated genes. Consequently, BCR::ABL1‐induced genes are especially sensitive to enhancer‐targeting CBP/P300‐inhibiting drugs, which is likely due to their enhancer‐regulation and especially high expression that we describe here as features of CBP/P300 inhibition‐sensitive genes. Our work further suggests that the addition of CBP/P300‐inhibiting drugs to current TKI treatments could benefit Ph+B‐ALL patients, especially TKI‐resistant patients and those from the high‐risk *IKZF1* ^PLUS^ subgroup. While CBP/P300 targeting could be perceived as too broad approach with likely side‐effects, it is successfully applied to other hematologic malignances [63] and by extension should be safe for Ph+B‐ALL as well. Indeed, unlike BET inhibitors, which clinical application is limited by thrombocytopenia and neutropenia as most common and severe (grade ≥3) adverse events [68], addition of Inobrodib to standard therapy of myeloma patients did not cause significant increase in frequency or severity of thrombocytopenia and neutropenia in a current trial [63]; and here, we show that Inobrodib does improve the efficacy of TKIs.

We also show that P300/CBP targeting acts in synergy with TKIs, especially in Ph+B‐ALL cells from *IKZF1* ^PLUS^ patients. The observed synergy is likely due to that enhancer targeting also suppresses genes that are crucial for Ph+B‐ALL but not induced by BCR::ABL1. An example of that would be *CDK6*, which is essential for Ph+B‐ALL [33] and enhancer‐driven (Figure 2D,I) but not sensitive to TKI treatment. *IKZF1* ^PLUS^ patients on the other hand, display loss of IKZF1 function, which can act as a transcriptional repressor and has been shown to negatively regulate *MYC* expression through suppression of its superenhancer [57]. As such, cells from *IKZF1* ^PLUS^ patients likely have elevated activity of enhancers usually suppressed by IKZF1 and thus could rely more on enhancer function, causing increased sensitivity to enhancer inhibition.

But how does BCR::ABL1 deregulate enhancer function and related transcriptional programs if BCR::ABL1 is neither a TF nor a nuclear protein? We identified STAT5, ETV5, and MYC as the key mediators, with the loss of each reducing H3K27ac levels at BCR::ABL1‐induced enhancers. However, in line with MYC being a central hub regulator in BCR::ABL1‐driven chronic myeloid leukemia (CML) [69], MYC also appeared most important in Ph+B‐ALL, while STAT5 and ETV5 instead displayed minor relevance, potentially linked to individual Ph+B‐ALL subgroups [60]. Nevertheless, most BCR::ABL1‐induced enhancers were regulated independent of MYC, STAT5, or ETV5.

While we specifically investigated BCR::ABL1‐induced enhancer regulation in Ph+B‐ALL, it is further important to note that this is not limited to Ph+B‐ALL but also occurs in BCR::ABL1‐driven CML cells; as supported here by us and previously by others that highlighted the relevance of enhancers in CML stem cells [69, 70]. Enhancer signatures do differentiate Ph+B‐ALL from CML cells, though, as enhancer activities differ between different cell types; and CML cells do show less sensitivity to enhancer targeting drugs compared to hypersensitive Ph+B‐ALL cells.

Lastly, as our study generated multiple datasets on enhancer location, 3D chromatin interactions, and CBP/P300 inhibition sensitivity, these could be of further use for others when studying a specific enhancer of interest with relevance in leukemia. While individual enhancers were not the prime focus of this study, it is important to mention that their validation by 3C assay and CRISPR interference should be a prerequisite for further study.

Together, our study provides important new insights into how BCR::ABL1 induces transcriptional reprogramming to promote Ph+B‐ALL. By investigating BCR::ABL1‐induced enhancer regulation, we discovered that BCR::ABL1‐regulated genes are highly sensitive to enhancer inhibition, and Ph+B‐ALL cells are subsequently hypersensitive to enhancer‐targeting drugs. Importantly, this and our results on combined treatment of Ph+B‐ALL cells with TKIs and CBP/P300 inhibitors/degraders thereby provide a basis for their potential future addition to current therapy.

## 4 Experimental/Methods Section

### 4.1 Cell Lines

All cell lines were originally obtained from the Deutsche Sammlung von Mikroorganismen und Zellkulturen (DSMZ, Germany), which validates its cell lines by STR analysis. For the Ph+B‐ALL cell lines predominantly used in this study, another re‐validation by STR analysis was performed at the Cell Science platform of the Francis Crick Institute (London, UK) at the end of the study, and cell lines were regularly tested for mycoplasma contamination. Research Resource Identifiers (RRID) of the lines used are: CVCL_1895 (TOM‐1), CVCL_0103 (SUP‐B15), CVCL_0181 (BV‐173), CVCL_1806 (AMO1), CVCL_1600 (NCI‐H929), CVCL_1334 (KMS‐12‐BM), CVCL_0004 (K‐562), CVCL_2091 (KCL‐22), CVCL_0388 (LAMA‐84), CVCL_0023 (A‐549), CVCL_0035 (PC‐3), CVCL_0063 (HEK293T), CVCL_0095 (SEM), CVCL_0093 (RS4;11), CVCL_1650 (REH), CVCL_0079 (697), CVCL_0590 (KASUMI‐2), CVCL_AQ30 (SMS‐SB), CVCL_4398 (OP9). TOM‐1/SUP‐B15 were purchased in 2019, AMO1/NCI‐H929/KMS‐12‐BM in 2023. A‐549, PC‐3, and OP9 were received from the Cell Science platform of the Francis Crick Institute (London, UK). All other cell lines were cryopreserved stocks obtained from the Müschen lab (Yale University) and purchased in 2003 from DSMZ. Human leukemia and myeloma cell lines were cultured in RPMI 1640 media supplemented with 1% penicillin/streptomycin, 1X Glutamax, and 20% FBS (all from Sigma) at 37°C in a humidified incubator with 5% CO_2_, while HEK293T cells were cultured in DMEM media + 1% penicillin/streptomycin, 1X Glutamax, and 20% FBS. OP9 cells were cultured as the leukemia cell lines above, but using aMEM media (Invitrogen). Adherent HEK293T cells were split using Trypsin (Sigma Aldrich) when reaching a confluency of 80%. A549 and PC3 cells were a kind gift of Dr Richard Burt (Crick Institute/Imperial) and cultured as described for HEK293T cells.

### 4.2 Patient Samples and Ethics

Human leukemia samples used in this research project were deposited into, stored in, and subsequently retrieved from the Imperial College Healthcare Tissue Bank (ICHTB). ICHTB is approved by NRES to release human material for research (12/WA/0196). A sub‐collection dedicated to this project was initiated (MEC_AR_16_030), and access to this sub‐collection for this project was granted. Healthy BM samples were provided as cryopreserved samples by The John Goldman Centre for Cellular Therapy of Hammersmith Hospital and collected under respective licenses or obtained from Cambridge Bioscience as bone marrow mononuclear cells. All patients gave specific informed consent for the use of surplus tissue. All samples were pseudo‐anonymised on receipt and given a study ID. For processing of primary B‐ALL samples, whole blood, bone marrow, or leukapheresis from these patients was separated by density gradient using centrifugation according to the manufacturer's protocol to retrieve mononuclear cells. Cells were then processed for ChIP‐Seq, cryopreservation, RNA extraction, etc. Purity of samples was checked by flow cytometry. Cytogenetic information to define if a sample was Ph+ or not was provided by the diagnostic lab of the Department of Haematology, Imperial College Healthcare NHS Trust. Ph+B‐ALL cells from patients were also expanded ex vivo on sub‐lethally irradiated (13.6 or 20 Gy) OP9 cells. After ∼20 days of co‐culture, cells were checked for viability and B‐ALL phenotype by flow cytometry and then split to new OP9‐coated plates for respective experiments on 24‐well plates or 10 cm dishes. For healthy controls, cryopreserved cells were thawed and either sorted by flow cytometry using anti‐human CD10 and CD19 antibodies or enriched by magnetic separation using the human B Cell Isolation Kit II and LS columns (Miltenyi Biotec), followed by RNA‐Seq or ChIP‐Seq analysis. MACS enrichment was validated by flow cytometry. Patient derived xenograft (PDX) samples used for HiChIP were generated as described in [71], and cells used to create them were collected from patients who gave informed consent and processed in compliance with the internal review boards of the Beckman Research Institute of City of Hope and all relevant ethical regulations. PDX samples used for ex vivo drug treatments (Figure S6B) were developed by Dr. Burt from primary patient ALL cells sourced from participants enrolled in the UKALL14 trial (NCT01085617).

### 4.3 In Vitro Transformation Using Murine B‐Cell Precursors and Ethics

Experiments using murine cells were performed under project license 70/8586 and in agreement with ASPA guidelines, regulations, and protocols approved by the Home Office UK. For all mouse experiments, cells were pre‐cultured in Iscove's Modified Dulbecco's Medium (IMDM) containing 20% FCS, 1% Penicillin/Streptomycin, 1X Glutamax, 50 µM 2‐mercaptoethanol (all from Sigma), and 10 ng/mL of recombinant mouse IL‐7 (Peprotech), followed by transduction with BCR::ABL1 encoding retrovirus or empty vector (EV) as control. Virus production was performed as described in Boulianne et al. [37] but using BCR::ABL1^p190^ or BCR::ABL1^p210^ encoding MIGR1 vectors, and respective EV. Viral transductions of B‐cell precursors (BCPs) from *Trp53bp1* ^−/−^ mice [72] were performed as previously described [37]. For C57BL/6 mice, cells were pre‐cultured for 5 days with mIL7 to enrich for BCPs, followed by two transductions as in [37]. For RNA‐Seq analysis, on day 1 after the second transduction, cells were sorted for GFP using flow cytometry (MA900 sorter, Sony), put back into culture, and harvested on day 3 or maintained at 0.5–2.5 x 10^6^ cells/mL and harvested at ∼ 50‐60 days post transduction. For H3K27ac ChIP‐Seq, no sort of GFP+ cells was performed. IL7‐induced enrichment of BCPs was monitored by flow cytometry, and viability and density were continuously monitored using trypan blue staining.

### 4.4 Flow Cytometry

For validation of human healthy or leukemic B‐cell precursors, the following antibodies were used as indicated in the figures: Anti‐CD10 (BioLegend, cl.HI10A), anti‐CD19 (BioLegend, cl.HIB19), anti‐CD179a (VpreB, BioLegend), and anti‐IgL (BioLegend). For staining of live/dead/apoptotic cells 7‐aminoactinomycin D (7‐AAD, Thermo Fisher Scientific) and Annexin‐V‐APC (BioLegend) were used. For cell cycle analysis, HOECHST 33342 dye (sigma) was used according to the manufacturer's instructions. Cells were then analysed by flow cytometry using a BD LSR‐Fortessa cytometer.

### 4.5 Monitoring Cell Viability and Proliferation Using Cell Titer Glo (CTG)

For cell lines and primary B‐ALL cells cultured without feeder layer cells, cells were seeded in 96‐well plates at 25,000 cells/well and treated with the drugs indicated. On day 2, a partial media change, adding new drugs and diluting cells 1:1 was performed. On day 4, cells were analysed using the Cell Titer Glo assay (Promega) and a TECAN plate reader. For primary B‐ALL cells cultured on OP9 cells, primary cells were first expanded and adapted to co‐culture for 3 weeks, by culturing them on 10 cm dishes with 0.75 x 10^6^ mitotically inactivated (20Gy) OP9 cells (OP9‐IR). CTG experiments were then performed on 24‐well plates containing 25,000 OP9‐IR cells and 0.4–0.8 x 10^6^ B‐ALL cells, depending on the availability of each sample, and the respective drugs added. Wells with OP9‐IR cells only were assessed as controls. On day 4 of treatment, B‐ALL cells were harvested for Cell Titer Glo (CTG) analysis by careful resuspension without disruption of OP9‐IR cells (4x gentle pipetting).

### 4.6 Monitoring Cell Viability by 7‐AAD and Annexin‐V Staining

Leukemia and myeloma cells were seeded in 12‐well plates at 0.5 x 10^6^ cells/mL and treated as indicated in the text and figure legends. For 5 day drug treatments, cells were split on day 2 or 3 (1:3) to dilute cells, replenish media, and add fresh drugs. On day 5 of treatment, cells were counted using Trypan blue and a haemocytometer, and remaining cells were stained for 7‐AAD (Thermo Fisher Scientific) and Annexin‐V‐APC (BioLegend) according to the manufacturer's instructions. Stained cells were analysed using an LSR‐Fortessa flow cytometer (BD). For adherent cells, 0.7 x 10^6^ HEK293T or A549 cells were seeded per well of a 6‐well plate, while PC3 cells were seeded at 0.6 x 10^6^ cells/well into a 12‐well plate. Cells were allowed to attach to the plate for 6 h before the start of drug treatment. Drug treatment and analysis were done as described above for suspension cells, but cells were detached using trypsin and split 1:5 for A549 and HEK293t cells. PC3 cells were just replated. For 1 or two day drug treatments, cells were directly stained using 7‐AAD (Thermo Fisher Scientific) and Annexin‐V‐APC (BioLegend) according to the manufacturer's instructions.

### 4.7 Western Blot

Western blot was performed as described in Boulianne et al. [37] using 4%–15% Mini‐PROTEAN TGX Precast Gels and the Mini‐PROTEAN blotting system (Bio‐Rad), but using the following antibodies: Anti‐phospho‐CRKL (#3181), anti‐H3K27ac (#4353), anti‐Histone 3 (#9715), anti‐Lamin B1 (#12586), anti‐beta‐Tubulin (#2128), anti‐HA‐tag (#3724), and anti‐STAT5A/B (#94205), phospho‐Stat5 (Tyr694) (#D47E7), anti‐STAT3 (#12640), anti‐STAT6 (#5397), anti‐P300 (#86377), anti‐ETV5 (#16274), phospho‐c‐Myc (Ser62) (#E1J4K) and c‐Myc/N‐Myc (#D3N8F) as primary antibodies (all from Cell Signaling), and goat anti‐rabbit HRP (R&D systems) as secondary antibody. For visualisation, Pierce ECL Western Blotting Substrate (Thermo Fisher) and a Chemidoc Touch Imager (Bio‐Rad) were used.

### 4.8 Drug Treatments

Ponatinib, Asciminib, dCBP‐1, Inobrodib (CCS1477), and MYC degrader 1 were obtained from Cambridge Biosciences / MedchemExpress. AK‐2292 [73] was from Cambridge Biosciences / MedchemExpress or Sigma Aldrich. A‐485 and PD0325901 were from Sigma Aldrich. SD‐436/AK‐1690 [74, 75] were kind gifts from Shaomeng Wang (University of Michigan). Drug treatments were performed for the times indicated in the text and Figure legends. For all drug experiments, 1000X stocks (drug in DMSO) were used and added in a 1:1000 manner. DMSO (Sigma) treatment was performed as a negative control for all experiments.

### 4.9 RNA Interference (RNAi)

ETV5 was depleted using a lentivirus containing TRCN0000013938‐encoding MISSION pLKO.1‐puro‐CMV‐TurboGFP plasmids (MERCK) and custom‐made 9–11 seed controls where nucleotides 9–11 of the shRNA seed sequence of TRCN0000013938 were mutated [76]. The 9–11 control plasmid was generated by first restoring the Age‐I site of TRCN0000013938‐pLKO.1‐puro‐CMV‐TurboGFP plasmids using the Q5 Site‐Directed Mutagenesis Kit (NEB) with GAAACACCGGtCGTGACACTT and GTCCTTTCCACAAGATATATAAAGC as primers, and then by replacing the original shRNA with the 9–11 mutated shRNA sequences CCGGGAGCGATACGAGAACAAATTTCTCGAGAAATTTGTTCTCGTATCGCTCTTTTTG and AATTCAAAAAGAGCGATACGAGAACAAATTTCTCGAGAAATTTGTTCTCGTATCGCTC. Lentivirus was generated as described in Pfeifer et al. [77]. Transduced cells were sorted for GFP at 3 or 4 days after transduction using a SONY MA900 and seeded as 5,000 cells/well into 96‐well plates using the sorter. Three days later, cells were collected, counted, and seeded at 1 x 10^6^ cells/mL into 12‐wells and treated with DMSO or 2.5 µM AK‐2292 for another 24 h before harvest for RNA‐Seq or ChIP‐Seq. For CTG analysis, three days after sorting, cells were kept in 96‐well plates but diluted 1:1 to add fresh media and DMSO or AK‐2292. Cells were then treated as described for CTG analysis above (1:1 dilution to refresh media and add drugs on day 2 of drug treatment, analysis by CTG on day 4).

### 4.10 RNA‐Seq, ChIP‐Seq, and ATAC‐Seq Library Preparation

Samples for RNA‐Seq were stored as snap‐frozen cell pellets, and RNA was extracted using the RNeasy mini kit (Qiagen). Total RNA was submitted to and processed for RNA‐Seq library preparation by Novogene (UK). ChIP‐Seq for H3K27ac was done as described in Boulianne et al. [37] using anti‐Histone H3 (acetyl K27) antibody – ChIP Grade (ab4729) (Abcam) and polyclonal rabbit IgG (isotype control) (ab171870) (Abcam), with the exception that initial cross‐linking was performed at RT. H3K27ac and respective IgG ChIP‐Seq was performed on 15 x 10^6^ or 1 x 10^6^ cells depending on cell availability but using the same protocol (i.e., experiments shown in Figure 5 were performed with 1 x 10^6^ cells) and using internal treatment controls for comparisons. ChIP‐Seq for MYC (D3N8F) and STAT5 (D206Y) (Cell Signalling Technologies) and respective IgG controls were performed using 50 x 10^6^ cells double‐crosslinked with 2 mM Disuccinimidyl Glutarate (DSG) followed by 1% formaldehyde. ChIP‐Seq libraries were generated from ChIP immunoprecipitated DNA using the NEBNext Ultra^TM^ II DNA Library Prep Kit for Illumina and NEBNext Multiplex Oligos for Illumina (NEB) and sequenced either at Novogene or at the in‐house sequencing facility of the MRC London Institute of Medical Sciences at Imperial College London. For ATAC‐Seq, samples were processed according to the Omni‐ATAC‐seq protocol (https://doi.org/10.1038/protex.2017.096) and similarly sequenced by Novogene. Sequenced read numbers and further information can be found in Supplementary Table 18.

### 4.11 PCHi‐C and HiChIP Library Generation

PCHi‐C was generated as previously described [78]. As a quality control step, 3C‐PCR and HindIII/NheI digestions were performed as described [41], with primers targeting the *MYC* locus (forward primer sequence: GGAGAACCGGTAATGGCAAA) and two known interaction sites (site e1 reverse primer sequence: TGCCTGATGGATAGTGCTTTC; site e2 reverse primer sequence: AAAATGCCCATTTCCTTCTCC). PCHi‐C libraries were sequenced by paired‐end (75 bp) sequencing at the BRC genomics facility at Imperial College London.

HiChIP was performed using the Arima Genomics HiChIP kit with the recommended protocol. H3K27ac (Active Motif #91193) was used for immunoprecipitation of libraries. HiChIP libraries were prepared using the Arima Genomics HiChIP library preparation with Swift Biosciences Accel‐NGS 2S Plus DNA library kit. HiChIP libraries were sequenced with PE (150 bp) sequencing at the in‐house sequencing facility of the Yale School of Medicine.

### 4.12 Bioinformatic Processing of NGS Libraries

For RNA‐Seq, ChIP‐Seq, and ATAC‐Seq libraries, raw fastq files were trimmed using Trim‐galore (v0.6.7) before further analysis. GRCh38 (hg38), or GRCm39 (mm39) reference genomes were used for alignment of reads for all human and mouse samples, respectively. For all analysis on R (v4.1.0), tidyverse (v1.3.1), and magrittr (v2.0.1) were loaded.

#### 4.12.1 RNA‐Seq

Salmon (v1.10.1) was used to quantify transcript count from RNA‐Seq. A full decoy index was generated using whole genome and transcript sequences obtained from the GENCODE genes database, with the hg38 (release 43) and mm39 (release 32) genomes, respectively. Trimmed fastq files were processed using Salmon (v1.10.1) with the following parameters: ‐ ‐gcbias, ‐ ‐writeUnmappedNames.

GenomicFeatures(v1.46.5) was used to generate transcript‐to‐gene objects in R, using GTF files obtained from the GENECODE gene database. The salmon quantification file was subsequently analysed using DESeq2 (v1.34.0). Briefly, tximport (v1.22.0) was used to import Salmon quantification files into R. DESeqDataSetFromTximport was used to process transcript count. As the human cell lines were inherently different in genome, this difference was factored in when using DESeq2 in our comparison between DMSO and ponatinib treatment. In DESeqDataSetFromTximport(), the following parameter was used in the “design” option: ∼condition+cell_line. For all other analyses with DESeq2, ∼condition was used in the “design” option. Subsequently, genes with low transcript counts below 5 were filtered out from the analysis using rowSums(counts()) >= 5. DESeq() and results() were used to determine differentially expressed genes (DEGs). Data generated by results() were filtered for DEGs with a p‐adjusted value < 0.05 and a log2 fold change > 1 or < −1.

To generate DEG expression heatmaps, rlog() transformation, followed by assay()[DEG,] (where DEG is a vector of DEGs) generated a matrix. The matrix was used to plot a heatmap with pheatmap (v1.0.12), where the scale = ″row″ option was added.

For principal component analysis (PCA) plots, plotPCA() was used with intgroup = c(“Treatment”) option. Scripts (Salmon + DESeq2) are available at GitHub at https://github.com/HanLengNg (refer to the ICL‐RNAseq_salmon and ICL‐DESeq2_script repositories, respectively).

#### 4.12.2 ChIP‐Seq

Trimmed fastq files were aligned to the human genome, hg38, using bowtie2 (v2.4.4), using the additional parameter: –no‐unal. SAM files generated by bowtie2 were converted to BAM format using samtools (v1.14) view ‐S ‐b. Bam files were processed using samtools fixmate ‐m, samtools sort ‐o, samtools markdup ‐r, samtools collate ‐o, and samtools sort ‐o again.

To generate a file for visualisation on a genome browser, the final bam files were processed by the deeptools (v3.5.1) suite. To generate bigwig files, bamCoverage was used with the following parameters: ‐ ‐normalizeUsing RPKM, ‐ ‐extendReads, ‐ ‐effectiveGenomeSize 2913022398 (for hg38), or 2654621783 (for mm39).

To determine peak enrichment across the genome, macs2 (v2.2.7.1) callpeak was used, with an IgG ChIP used as the input control. The following parameters were used: ‐q 0.05, ‐B, ‐ ‐SPMR, ‐ ‐broad (only for H3K27ac ChIP), and ‐g hs (for hg38) or mm (for mm39).

To determine reads within specific region, the Homer (v4.11) package suite was used. The Homer suit was used to create tag directories using makeTagDirectory, with the added parameter: ‐ ‐keepAll. The script is available at GitHub at https://github.com/HanLengNg (refer to the ICL‐ChIPseq repository).

#### 4.12.3 ATAC‐Seq

Trimmed fastq files were aligned to the human genome, hg38, using bowtie2 (v2.4.4) with the same parameters as for ChIP‐Seq. Similarly, alignment SAM files were processed with samtools in the identical method as for ChIP‐Seq. To generate bigwig files for viewing on a genome browser, bamCoverage (from the deeptools package) was used with the same parameters as for ChIP‐Seq, except for the additional parameter of –extendReads.

ATAC‐Seq peaks were called using macs2 as for ChIP but without input control and not using –broad.

#### 4.12.4 PCHi‐C

Libraries were processed in the same method as previously described [78]. Raw fastq data were processed by HiCUP (v0.7.3) with the following parameters set in the configuration file: bowtie2, longest di‐tag length: 850, shortest di‐tag length: 100. The intermediate file generated by HiCUP was further processed in R using CHiCAGO (v1.18.0) and processed as described in Freire‐Pritchett et al. [79] to generate interaction files and scores. Interaction files generated by CHiCAGO were visualised on the WashU epigenome browser https://epigenomegateway.wustl.edu/browser/. The makePeakMatrix.R pipeline was used to generate a matrix file of all interactions in all PCHi‐C samples.

### 4.13 Computational Analyses

#### 4.13.1 Gene Promoters 2 Kb Flanking Region

Gene promoters were obtained from the GENCODE gene database for hg38 and mm39. Start coordinates of gene transcriptional start sites were obtained from GTF files, and the 2 kb region flanking (both up‐ and downstream) each transcriptional start site was obtained using bedtools slop ‐b 1000.

#### 4.13.2 H3K27ac ChIP‐Seq Heatmap

Non‐promoter peaks were obtained using bedtools intersect between H3K27ac peaks called by macs2 with gene promoter coordinates. Homer package was used to determine differential peaks between DMSO‐ and Ponatinib‐treated samples using the getDifferentialPeaks command and the H3K27ac peaks called by macs2, with the parameters: ‐P 0.05, ‐F 2, ‐size 500, with ‐rev either added or not, to determine peaks gained or lost with Ponatinib treatment, respectively. H3K27ac peak genomic coordinates that were within 1 kb of each other were merged together using bedtools merge, with the added parameter: ‐d 1000. To determine the consensus differential peak region between samples, bedtools multiinter was used, where the region overlapped by all three samples was filtered. To get the normalised peak score within the consensus region, Homer getPeakTags was used with the consensus differential peaks. Scores generated by getPeakTags were used to plot a heatmap in R, using dplyr to stack peaks score and converted to a matrix for plotting with pheatmap, with the parameters: scale = “row”, cluster_cols = FALSE, cluster_rows = FALSE.

#### 4.13.3 H3K27ac PCA Plots

H3K27ac peaks called by macs2 were split into either promoter or non‐promoter using bedtools intersect. DiffBind (v3.4.11) was used to generate PCA plots in R. In sum, a table is required containing macs2 peaks in bed format, together with the bam files generated from ChIP‐Seq analysis. The table was used in R with dba() and processed by dba.count(). The data was further normalised with dba.normalize() with the added options: normalize = DBA_NORM_LIB, and library = DBA_LIBSIZE_PEAKREADS. Finally, PCA plots were generated using dba.plotPCA() with the option: attributes = DBA_TREATMENT.

#### 4.13.4 H3K27ac DiffBind Analysis

Differential H3K27ac peaks were identified for the experimental groups defined in the text/figures using R with r‐tidyverse and Bioconductor‐diffbind packages and H3K27ac ChIP‐Seq bam files and MACS2‐defined peak bed files from n = 2 sets of experiments as input. Outputs were filtered for downregulated peaks, and p<0.05 and then compared for overlaps using bedtools intersect.

#### 4.13.5 PCHi‐C Analysis

Interaction data generated by CHiCAGO were used to determine overlap with H3K27ac peaks for both the bait (promoter) and other end (OE). For this, bedtools intersect was used to determine promoters and OE that overlap with H3K27ac peaks. In doing so, we were able to determine active promoters and inactive promoters by the presence or absence of H3K27ac, respectively. Bedtools groupby was used to group PCHi‐C interactions by either the presence of at least of one H3K27ac‐overlapping OE, or the complete absence of H3K27ac‐overlapping OE. As such, using bedtools groupby, we were able to classify four different types of interactions: 1) active promoter to active OE interaction(s); 2) active promoter to inactive OE interaction(s); 3) inactive promoter to active OE interaction(s); 4) inactive promoter to inactive OE interaction(s). Further, bedtools intersect was used to determine if any of the OE interactions overlap with any gene promoter. Together with bedtools groupby from the bait end, we were able to determine each bait interacting with the OE to be either one of three possible situations: enhancer‐promoter interactions (EPIs), promoter‐promoter interactions (PPIs), or a combination of EPIs and PPIs.

To determine PCHi‐C interactions and changes with Ponatinib treatment, RNA‐Seq data were utilised. Prior to analysis, active genes were filtered for a normalised transcript count of 10 or more using dplyr::filter(). By comparing between healthy human B‐cell precursors and Ph+ samples, Ph‐driven DEGs were identified from DESeq2. Additionally, TKI‐sensitive DEGs were determined by DESeq2 by comparing between DMSO‐ and Ponatinib‐treated Ph+ samples. Using both sets of DEGs, we were able to identify one of four groups of genes: 1) Ph‐upregulated genes that are not TKI‐sensitive; 2) Ph‐upregulated and TKI‐sensitive (down‐regulated) genes; 3) TKI‐sensitive (down‐regulated) and not deregulated by Ph; 4) cell line‐specific genes that are not in one of the three previous groups.

Using the list of all TKI‐sensitive genes, we were able to determine TKI‐sensitive interactions using dplyr::filter(). Additionally, we were able to separate the interactions into either EPIs, PPIs, or a mixture of EPIs and PPIs. For our analysis, all EPIs were grouped together, and CHiCAGO scores were determined based on their promoter baits and plotted for each promoter in samples treated with DMSO or TKI using GraphPad Prism (v9). Additionally, using the H3K27ac signal at both ends of each interaction, changes in H3K27ac enrichment were determined for interactions in DMSO‐treated samples and the corresponding interaction (or loss of interaction) after Ponatinib treatment.

From each sample, we were able to obtain a list of active promoters to active enhancer interactions (active EPIs) with dplyr::filter(). The lists of active EPIs were used to define a set of core Ph+ interactions. Using the number of interactions for each sample, a Venn diagram was generated in R using draw.triple.venn(). Additionally, the matrix generated by makePeakMatrix.R was filtered for only the core Ph+ interaction set and used to plot a hierarchical dendrogram in R. The matrix was transformed, t(), followed by using the dist() function with the option: method = “euclidean” and used with the hclust() function with the option: method = “average”. The same matrix was also used to generate a PCA plot. First, the matrix was transformed t(), followed by prcomp(), and finally plotted using autoplot().

#### 4.13.6 Barnett et al. PCHi‐C Analysis

PCHi‐C data from Barnett et al. were obtained from GSE211631. Raw fastq data were processed following the snakemake pipeline written to process Arima PCHiC‐generated sequencing reads (https://github.com/insilicoconsulting/snake‐chic) with the hg38 reference genome. The HiCUP digest file, hg38 CHiCAGO input 3kb rmap file, and hg38 CHiCAGO input 3kb baitmap file were obtained from Arima Genomics to perform the PCHi‐C analysis. With the Rds file generated from the pipeline, the makePeakMatrix.R command from the CHiCAGO package was used to generate a peak matrix of interactions from all samples. Using R tidyverse inner_join(), a list of core Ph interactions was identified using SUP‐B15 and patient 101 BEDPE files. As we were unable to obtain H3K27ac ChIP‐Seq and ATAC‐Seq data for both SUP‐B15 and patient 101 from Barnett et al., H3K27ac ChIP‐Seq and ATAC‐Seq for SUP‐B15 generated here were used instead. To determine interactions containing H3K27ac, or open chromatin (by ATAC‐Seq) on both ends of each chromatin loop, bedtools pairToBed was used with the parameter: ‐type both. Using the list of H3K27ac or ATAC Ph core interactions, PCHi‐C scores were extracted from the peak matrix for all samples with R tidyverse semi_join(). The filtered peak matrices were processed in R with t(), followed by prcomp(), and lastly with autoplot() to generate the PCA plots.

#### 4.13.7 A‐485/dCBP‐1 and PCHi‐C Analysis

DESeq2 was utilised to determine DEGs from cells treated with A‐485 or dCBP‐1. Using the PCHi‐C interaction data, A‐485 and dCBP‐1 DEGs were separated into either having EPI only, PPI only, a mixture of both EPIs and PPIs, and other genes with no interaction, and results were analysed and visualised using GraphPad PRISM.

#### 4.13.8 HiChIP Analysis

The MAPS pipeline generated a BEDPE file of both ends of interactions, where a raw read count is calculated from the HiChIP fastq files, as well as different expected contact frequency calculated for interaction clusters. For each sample, the read count for each interaction was normalised to the library size of per 10 million reads to get an interaction score. Additionally, each interaction had different expected contact frequency for the cluster, and as such, unique interactions were filtered for downstream analysis using the unique() function in R. Using the filtered list of unique interactions for all samples, we were able to define three types of interactions: 1) promoter‐promoter interactions; 2) promoter‐promoter interactions; 3) enhancer‐enhancer interactions. These interactions were determined using bedtools intersect with known gene promoter coordinates for both ends of the interactions. For our analysis, we examined how the samples clustered from each of the three types of interactions: promoter‐promoter, promoter‐enhancer, and enhancer‐enhancer. Hierarchical clustering was performed on R using t(), dist(), and hclust() for downstream PCA analysis. Similar to PCHi‐C PCA analysis, the data were processed in R using t(), prcomp(), and autoplot() in the respective order.

#### 4.13.9 Target RNA‐Seq Analysis

Publicly available RNA‐seq data from the TARGET study (Therapeutically Applicable Research to Generate Effective Treatments initiative, phs000463 and phs000464, https://www.cancer.gov/ccg/research/genome‐sequencing/target) were downloaded for B‐ALL with diagnosis known for either Ph+ (n = 6), or KMT2A‐r (n = 4). The data used for this analysis are available at the Genomic Data Commons (https://portal.gdc.cancer.gov). DESeq2 was used to identify DEGs between Ph+ and KMT2A‐r B‐ALL.

#### 4.13.10 HiChIP and RNA‐Seq Combined Analysis

From the RNA‐Seq analysis of Ph+ and KMT2A‐r B‐ALL (TARGET) samples, DEGs were used for HiChIP samples. Using dplyr::filter(), DEGs were used to filter for HiChIP interactions that overlap with the promoter at either end of the HiChIP interactions. From the filtered interactions, an average number of interactions was counted for each gene across all samples analysed. The average number of interactions was further processed to generate a log2 fold change value to define if a gene had more interactions in the Ph+ B‐ALL or KMT2A‐r B‐ALL samples. Similarly, RNA‐Seq log2 fold changes for these DEGs were filtered and combined with the average HiChIP log2 fold change interaction in a table in R. To generate a trend line for the graph, in ggplot, the function geom_smooth was added with the following option: method = “gam”, formula = y ∼ s(x).

#### 4.13.11 DepMap Dependency Analysis

DepMap compound screen values were obtained via the DepMap portal / Data Explorer at https://depmap.org/portal/interactive/ for the cancer types indicated and plotted as indicated via GraphPad PRISM.

#### 4.13.12 Gene Ontology (GO) Analysis

GO analyses were performed using R (for Figure S2K) or using the Gene Ontology Resource platform at https://geneontology.org (for Figure 4K). For GO using R, Ensembl gene IDs were converted to symbols in R and were further analysed by Enrichr to obtain Biological Process enrichment figures. Ensembl gene ID lists for enhancer – promoter interaction genes only (EPIonly), promoter – promoter interaction genes only (PPIonly) and enhancer – promoter with promoter – promoter interactions genes (EPIPPI) were obtained and loaded in R using read.table() with the readr package (version 2.1.5). The Ensembl gene stable IDs were converted into gene symbols with org.Hs.eg.db package (version 3.19.1) and clusterProfiler package (version 4.12.0) with the added options: keytype = “ENSEMBL”, column = “SYMBOL”. Next, na.omit() was used to remove gene symbols with NAs from the list, before uploading the list of genes to the Enrichr website (available at: https://maayanlab.cloud/Enrichr/) to obtain a GO biological process enrichment table. Finally, the bar charts were generated based on the table by plotEnrich() using enrichR package (version 3.2) with the added options: showTerms = 15, numChar = 80, y = “Count”, orderBy = “P.value”.

### 4.14 Data Availability

Raw and processed data generated here can be found at Gene Expression Omnibus (GEO) under accession numbers GSE279594 (RNA‐Seq), GSE279643 (PCHi‐C), GSE279644 (ChIP‐Seq), GSE279589 (ATAC‐Seq) and GSE301240 (HiChIP).

### 4.15 Statistics

For NGS data processing and analysis, raw data for RNA‐seq and ChIP‐seq were trimmed for sequencing adaptors with Trim Galore before performing downstream analyses. Sequencing statistics are presented in Table S18. For RNA‐Seq, DESeq2 in R was used to detect DEGs, which are defined with log2 fold change greater or less than 1, with p‐adjusted values less than 0.05. To generate the heatmaps (Figure 1B–D), DESeq2 normalised data were transformed with rlog() and plotted with the pheatmap package. For ChIP‐seq analyses, differential non‐promoter H3K27ac peaks for heatmaps (Figures 1B–D and 4B,C; Figures S4A,B) were calculated using bedtools intersect. Comparisons were made from the reference of DMSO‐treated samples, where the option ‐u or ‐v was used with bedtools intersect to define DMSO and drug‐treatment, respectively. Using the differentially defined non‐promoter H3K27ac genomic regions, Homer getPeakTags was used to get a count of reads per 10 million total reads within the regions. Lastly, pheatmap was used to plot the normalized counts. For PCHi‐C, the CHiCAGO package in R was used to define chromatin interactions, with the default CHiCAGO score of 5 to represent significant interactions [79]. For HiChIP analysis, significant interactions were analyzed with the Arima Genomics MAPS pipeline (https://github.com/HuMingLab/MAPS) with the default parameters. As H3K27ac was targeted for HiChIP, all significant interactions contained H3K27ac enrichment on at least one end of each chromatin loop. RNA‐Seq PCA plots were generated with plotPCA() in R on rlog transformed data. HiChIP PCA plots were generated by transformation with prcomp(), before plotting the data with autoplot(). H3K27ac PCA plots were generated with the DiffBind package in R. All libraries used for analysis in Figure 3A were normalized to library size and for defined regions (either promoter or non‐promoter) with the dba.normalize() command of DiffBind and plotted with dba.plotPCA.

For single comparisons, statistical analyses were mostly performed using unpaired student's t test and GraphPad PRISM 9/10 or R, with sample sizes indicated in the figure legends, data presented as mean ± SD or SEM, and P values indicating the statistical significance shown in the figures. Exceptions were Figure 4F–H and Supplementary Figure 5D, where a paired two‐tailed student's *t*‐test was performed and plots generated with GraphPad PRISM 9/10, with data presented in Figure 4F‐H as dots with overlapping box‐and‐whisker plots. Besides, the following pre‐processing was performed for some analyses: For Figures 2G–I and 4I–K and Figure S2N,O, data from n = 2 experiments of TOM‐1 and SUP‐B15 cells were merged. To allow joint comparison of selected genes despite cell line‐specific differences in expression levels in Figure 2I and Figure S2N, RNA‐Seq expression values were further normalized to average DMSO values. Likewise, for the analysis of viability and relative cell numbers by Figure 6E, values were normalized to average DMSO values. To demonstrate the effect of the addition of P300/CBP inhibitors to TKI treatment of Ph+B‐ALL cells in Figure 6H, relative numbers of live cells were normalized to those of ‘TKI only’ treated cells for each experiment.

For synergy score analysis by *SynergyFinder* in Figure 6G and Supplementary Figure 6A, combined treatment was defined by scores larger than 10 as likely synergistic, by scores from −10 to 10 as likely additive, and by scores of less than −10 as likely antagonistic.

For GO analyses in Figure 4K and Figure S2K, only enriched pathways with P<0.05 are analyzed, with pathways shown in Figure 4K passing P = 0.0002 and FDR<0.05.

## Author Contributions

H.L.N. performed most experiments, including PCHi‐C, HiChIP, ChIP‐Seq, RNA‐Seq, and respective bioinformatic analyses. T.L.G. performed RNA‐Seq and ChIP‐Seq experiments, RNAi, bioinformatic RNA‐Seq analyses, and degron experiments not included in the final manuscript. J.Z. performed GO analysis and ChIP‐Seq. M.E.R. helped with processing and bioinformatic analyses of HiChIP data and RNA‐Seq of mouse BCPs. K.N.C. helped with the processing of PDX samples for HiChIP. V.M. and M.S. helped with design and guidance for PCHi‐C. O.D. performed ATAC‐Seq, not included in this manuscript, and assisted with manuscript writing. N.T.C. provided guidance on degron/dTAG experiments not included in this manuscript, on bioinformatic analysis of ChIP‐Seq data, and on the manuscript. K.H. helped with murine RNA‐Seq experiments. L.S. helped with HiChIP analyses. A.J.I. assisted with patient samples. R.B. provided PDX cells for drug treatment experiments. G.J. helped with STAT degrader experiments. S.W. provided STAT3 and 6 degraders [74, 75]. M.M. supported HiChIP experiments. N.F. performed Western blots, flow cytometry, RNAi, degrader experiments, dTAG knock‐in generation not included here, mouse work, designed the study, and wrote the manuscript.

## Conflicts of Interest

The authors declare no conflicts of interest.

## Supporting information




**Supporting File 1**: advs74523‐sup‐0001‐SuppMat.pdf.

**Supporting File 2**: advs74523‐sup‐0002‐Tables.xlsx.

## Data Availability

All NGS data generated here is available at GEO under accession numbers GSE279589, GSE279594, GSE279643, GSE279644, GSE301240. Detailed information on protocols, methods and reagents can be found in the Methods section of our study. Any tools generated here (plasmids, cell lines etc.) will be provided on request.

## References

[1] R. C. Harvey , C. G. Mullighan , X. Wang , et al., “Identification of Novel Cluster Groups in Pediatric High‐Risk B‐Precursor Acute Lymphoblastic Leukemia with Gene Expression Profiling: Correlation with Genome‐Wide DNA Copy Number Alterations, Clinical Characteristics, and Outcome,” Blood 116 (2010): 4874–4884, 10.1182/blood-2009-08-239681. 20699438 PMC3321747

[2] Z. Gu , M. L. Churchman , K. G. Roberts , et al., “PAX5‐Driven Subtypes of B‐Progenitor Acute Lymphoblastic Leukemia,” Nature Genetics 51 (2019): 296–307, 10.1038/s41588-018-0315-5. 30643249 PMC6525306

[3] C. L. Jensen , L.‐F. Chen , T. Swigut , et al., “Long‐Range Regulation of Transcription Scales with Genomic Distance in a Gene‐Specific Manner,” Molecular Cell 85 (2025): 347–361, 10.1016/j.molcel.2024.10.021. 39626660 PMC11741922

[4] J. M. Alexander , J. Guan , B. Li , et al., “Live‐Cell Imaging Reveals Enhancer‐Dependent Sox2 Transcription in the Absence of Enhancer Proximity,” Elife 8 (2019): 41769, 10.7554/eLife.41769. PMC6534382 31124784

[5] N. S. Benabdallah , I. Williamson , R. S. Illingworth , et al., “Decreased Enhancer‐Promoter Proximity Accompanying Enhancer Activation,” Molecular Cell 76 (2019): 473–484, 10.1016/j.molcel.2019.07.038. 31494034 PMC6838673

[6] J.‐S. Roe , C.‐I. Hwang , T. D. D. Somerville , et al., “Enhancer Reprogramming Promotes Pancreatic Cancer Metastasis,” Cell 170 (2017): 875–888, 10.1016/j.cell.2017.07.007. 28757253 PMC5726277

[7] V. Poli , L. Fagnocchi , A. Fasciani , et al., “MYC‐Driven Epigenetic Reprogramming Favors the Onset of Tumorigenesis by Inducing a Stem Cell‐Like state,” Nature Communications 9 (2018): 1024, 10.1038/s41467-018-03264-2. PMC5844884 29523784

[8] M. Bi , Z. Zhang , Y.‐Z. Jiang , et al., “Enhancer Reprogramming Driven by High‐Order Assemblies of Transcription Factors Promotes Phenotypic Plasticity and Breast Cancer Endocrine Resistance,” Nature Cell Biology 22 (2020): 701–715, 10.1038/s41556-020-0514-z. 32424275 PMC7737911

[9] S. Gröschel , M. A. Sanders , R. Hoogenboezem , et al., “A Single Oncogenic Enhancer Rearrangement Causes Concomitant EVI1 and GATA2 Deregulation in Leukemia,” Cell 157 (2014): 369–381, 10.1016/j.cell.2014.02.019. 24703711

[10] L. E. Montefiori , S. Bendig , Z. Gu , et al., “Enhancer Hijacking Drives Oncogenic BCL11B Expression in Lineage‐Ambiguous Stem Cell Leukemia,” Cancer Discovery 11 (2021): 2846–2867, 10.1158/2159-8290.CD-21-0145. 34103329 PMC8563395

[11] B. J. Abraham , D. Hnisz , A. S. Weintraub , et al., “Small Genomic Insertions Form Enhancers That Misregulate Oncogenes,” Nature Communications 8 (2017): 14385, 10.1038/ncomms14385. PMC5309821 28181482

[12] M. R. Mansour , B. J. Abraham , L. Anders , et al., “An Oncogenic Super‐Enhancer Formed through Somatic Mutation of a Noncoding Intergenic Element,” Science 346 (2014): 1373–1377, 10.1126/science.1259037. 25394790 PMC4720521

[13] P. Gou and W. Zhang , “Protein Lysine Acetyltransferase CBP/p300: a Promising Target for Small Molecules in Cancer Treatment,” Biomedicine & Pharmacotherapy 171 (2024): 116130, 10.1016/j.biopha.2024.116130. 38215693

[14] T. Shorstova , W. D. Foulkes , and M. Witcher , “Achieving Clinical Success with BET Inhibitors as Anti‐Cancer Agents,” British Journal of Cancer 124 (2021): 1478–1490, 10.1038/s41416-021-01321-0. 33723398 PMC8076232

[15] T. Creasey , E. Barretta , S. L. Ryan , et al., “Genetic and Genomic Analysis of Acute Lymphoblastic Leukemia in Older Adults Reveals a Distinct Profile of Abnormalities: Analysis of 210 Patients from the UKALL14 and UKALL60+ Clinical Trials,” Haematologica 107 (2022): 2051–2063, 10.3324/haematol.2021.279177. 34788984 PMC9425332

[16] R. Foà , R. Bassan , L. Elia , et al., “Long‐Term Results of the Dasatinib‐Blinatumomab Protocol for Adult Philadelphia‐Positive ALL,” Journal of Clinical Oncology 42 (2024): 881–885, 10.1200/JCO.23.01075. 38127722 PMC10927329

[17] M. Stanulla , E. Dagdan , M. Zaliova , et al., “IKZF1plus Defines a New Minimal Residual Disease–Dependent Very‐Poor Prognostic Profile in Pediatric B‐Cell Precursor Acute Lymphoblastic Leukemia,” Journal of Clinical Oncology 36 (2018): 1240–1249, 10.1200/JCO.2017.74.3617. 29498923

[18] C. L. Sawyers , “The Role of Myc in Transformation by BCR‐ABL,” Leukemia & Lymphoma 11, no. Sup1 (1993): 45–46, 10.3109/10428199309047862. 8251915

[19] N. Sharma , V. Magistroni , R. Piazza , et al., “BCR/ABL1 and BCR Are under the Transcriptional Control of the MYC Oncogene,” Molecular Cancer 14 (2015): 132, 10.1186/s12943-015-0407-0. 26179066 PMC4504180

[20] V. Minieri , M. De Dominici , P. Porazzi , et al., “Targeting STAT5 or STAT5‐Regulated Pathways Suppresses Leukemogenesis of Ph+ Acute Lymphoblastic Leukemia,” Cancer Research 78 (2018): 5793–5807, 10.1158/0008-5472.CAN-18-0195. 30154155 PMC7100125

[21] S. Shojaee , R. Caeser , M. Buchner , et al., “Erk Negative Feedback Control Enables Pre‐B Cell Transformation and Represents a Therapeutic Target in Acute Lymphoblastic Leukemia,” Cancer Cell 28 (2015): 114–128, 10.1016/j.ccell.2015.05.008. 26073130 PMC4565502

[22] B. Kharabi Masouleh , H. Geng , C. Hurtz , et al., “Mechanistic Rationale for Targeting the Unfolded Protein Response in Pre‐B Acute Lymphoblastic Leukemia,” Proceedings of the National Academy of Sciences 111 (2014): E2219–2228, 10.1073/pnas.1400958111. PMC4040579 24821775

[23] I. Sanchez‐Garcia and G. Grutz , “Tumorigenic Activity of the BCR‐ABL Oncogenes Is Mediated by BCL2,” Proceedings of the National Academy of Sciences 92 (1995): 5287–5291, 10.1073/pnas.92.12.5287. PMC41679 7777499

[24] I. Sanchez‐Garcia and D. Martin‐Zanca , “Regulation of Bcl‐2 Gene Expression by BCR‐ABL Is Mediated by Ras,” Journal of Molecular Biology 267 (1997): 225–228, 10.1006/jmbi.1996.0779. 9096220

[25] R. P. de Groot , J. A. Raaijmakers , J. W. Lammers , and L. Koenderman , “STAT5‐Dependent CyclinD1 and Bcl‐xL Expression in Bcr‐Abl‐Transformed Cells,” Molecular Cell Biology Research Communications 3 (2000): 299–305, 10.1006/mcbr.2000.0231. 10964754

[26] S. Dumon , S. C. R. Santos , F. Debierre‐Grockiego , et al., “IL‐3 Dependent Regulation of Bcl‐xL Gene Expression by STAT5 in a Bone Marrow Derived Cell Line,” Oncogene 18 (1999): 4191–4199, 10.1038/sj.onc.1202796. 10435632

[27] F. Gesbert and J. D. Griffin , “Bcr/Abl Activates Transcription of theBcl‐X Gene through STAT5,” Blood 96 (2000): 2269–2276, 10.1182/blood.V96.6.2269. 10979976

[28] D. A. Frank and L. Varticovski , “BCR/Abl Leads to the Constitutive Activation of Stat Proteins, and Shares an Epitope with Tyrosine Phosphorylated Stats,” Leukemia 10 (1996): 1724–1730. 8892675

[29] N. Carlesso , D. A. Frank , and J. D. Griffin , “Tyrosyl Phosphorylation and DNA Binding Activity of Signal Transducers and Activators of Transcription (STAT) Proteins in Hematopoietic Cell Lines Transformed by Bcr/Abl,” The Journal of experimental medicine 183 (1996): 811–820, 10.1084/jem.183.3.811. 8642285 PMC2192351

[30] R. L. Ilaria and R. A. Van Etten , “P210 and P190 Induce the Tyrosine Phosphorylation and DNA Binding Activity of Multiple Specific STAT Family Members,” Journal of Biological Chemistry 271 (1996): 31704–31710, 10.1074/jbc.271.49.31704. 8940193

[31] R. C. Nayak , S. Hegde , M. J. Althoff , et al., “The Signaling Axis atypical Protein Kinase C λ/ι‐Satb2 Mediates Leukemic Transformation of B‐Cell Progenitors,” Nature Communications 10 (2019): 46, 10.1038/s41467-018-07846-y. PMC6320370 30610188

[32] K. Kollmann , G. Heller , R. G. Ott , et al., “c‐JUN Promotes BCR‐ABL–induced Lymphoid Leukemia by Inhibiting Methylation of the 5′ Region of Cdk6,” Blood 117 (2011): 4065–4075, 10.1182/blood-2010-07-299644. 21300982

[33] M. De Dominici , P. Porazzi , A. R. Soliera , et al., “Targeting CDK6 and BCL2 Exploits the “MYB Addiction” of Ph+ Acute Lymphoblastic Leukemia,” Cancer Research 78 (2018): 1097–1109, 10.1158/0008-5472.CAN-17-2644. 29233926 PMC5815914

[34] I. Menzl , T. Zhang , A. Berger‐Becvar , et al., “A Kinase‐Independent Role for CDK8 in BCR‐ABL1+ Leukemia,” Nature Communications 10 (2019): 4741, 10.1038/s41467-019-12656-x. PMC6802219 31628323

[35] M. Buchner , E. Park , H. Geng , et al., “Identification of FOXM1 as a Therapeutic Target in B‐Cell Lineage Acute Lymphoblastic Leukaemia,” Nature Communications 6 (2015): 6471, 10.1038/ncomms7471. PMC4366523 25753524

[36] K. Behrens , N. Brajanovski , Z. Xu , et al., “ERG and c‐MYC Regulate a Critical Gene Network in BCR::ABL1‐Driven B Cell Acute Lymphoblastic Leukemia,” Science Advances 10 (2024): adj8803, 10.1126/sciadv.adj8803. PMC10923517 38457494

[37] B. Boulianne , M. E. Robinson , P. C. May , et al., “Lineage‐Specific Genes Are Prominent DNA Damage Hotspots during Leukemic Transformation of B Cell Precursors,” Cell Reports 18 (2017): 1687–1698, 10.1016/j.celrep.2017.01.057. 28199841 PMC5318656

[38] H. Abdelrasoul , A. Vadakumchery , M. Werner , et al., “Synergism between IL7R and CXCR4 Drives BCR‐ABL Induced Transformation in Philadelphia Chromosome‐Positive Acute Lymphoblastic Leukemia,” Nature Communications 11 (2020): 3194, 10.1038/s41467-020-16927-w. PMC7314847 32581241

[39] A. Hoelbl , C. Schuster , B. Kovacic , et al., “Stat5 is Indispensable for the Maintenance of Bcr/Abl ‐Positive Leukaemia,” EMBO Molecular Medicine 2 (2010): 98–110, 10.1002/emmm.201000062. 20201032 PMC2906698

[40] F. Klein , N. Feldhahn , L. Harder , et al., “The BCR‐ABL1 Kinase Bypasses Selection for the Expression of a Pre–B Cell Receptor in Pre–B Acute Lymphoblastic Leukemia Cells,” The Journal of Experimental Medicine 199 (2004): 673–685, 10.1084/jem.20031637. 14993251 PMC2213306

[41] S. Schoenfelder , B. M. Javierre , M. Furlan‐Magaril , S. W. Wingett , and P. Fraser , “Promoter Capture Hi‐C: High‐Resolution, Genome‐Wide Profiling of Promoter Interactions,” Journal of Visualized Experiments 28 (2018): 57320, 10.3791/57320. PMC6102006 30010637

[42] B. Mifsud , F. Tavares‐Cadete , A. N. Young , et al., “Mapping Long‐Range Promoter Contacts in human Cells with High‐Resolution Capture Hi‐C,” Nature Genetics 47 (2015): 598–606, 10.1038/ng.3286. 25938943

[43] K. R. Barnett , R. J. Mobley , J. D. Diedrich , et al., “Epigenomic Mapping Reveals Distinct B Cell Acute Lymphoblastic Leukemia Chromatin Architectures and Regulators,” Cell Genomics 3 (2023): 100442, 10.1016/j.xgen.2023.100442. 38116118 PMC10726428

[44] B. M. Javierre , O. S. Burren , S. P. Wilder , et al., “Lineage‐Specific Genome Architecture Links Enhancers and Non‐Coding Disease Variants to Target Gene Promoters,” Cell 167 (2016): 1369–1384, 10.1016/j.cell.2016.09.037. 27863249 PMC5123897

[45] P. Freire‐Pritchett , S. Schoenfelder , C. Várnai , et al., “Global Reorganisation of Cis‐Regulatory Units Upon Lineage Commitment of human Embryonic Stem Cells,” Elife 6 (2017): 21926, 10.7554/eLife.21926. PMC5407860 28332981

[46] T. Narita , S. Ito , Y. Higashijima , et al., “Enhancers Are Activated by p300/CBP Activity‐Dependent PIC Assembly, RNAPII Recruitment, and Pause Release,” Molecular Cell 81 (2021): 2166–2182, 10.1016/j.molcel.2021.03.008. 33765415

[47] S. Sungalee , Y. Liu , R. A. Lambuta , et al., “Histone Acetylation Dynamics Modulates Chromatin Conformation and Allele‐Specific Interactions at Oncogenic Loci,” Nature Genetics 53 (2021): 650–662, 10.1038/s41588-021-00842-x. 33972799

[48] L. M. Lasko , C. G. Jakob , R. P. Edalji , et al., “Discovery of a Selective Catalytic p300/CBP Inhibitor That Targets Lineage‐Specific Tumours,” Nature 550 (2017): 128–132, 10.1038/nature24028. 28953875 PMC6050590

[49] R. Vannam , J. Sayilgan , S. Ojeda , et al., “Targeted Degradation of the Enhancer Lysine Acetyltransferases CBP and p300,” Cell Chemical Biology 28 (2021): 503–514, 10.1016/j.chembiol.2020.12.004. 33400925

[50] S. J. Hogg , O. Motorna , L. A. Cluse , et al., “Targeting Histone Acetylation Dynamics and Oncogenic Transcription by Catalytic P300/CBP Inhibition,” Molecular Cell 81 (2021): 2183–2200, 10.1016/j.molcel.2021.04.015. 34019788 PMC8183601

[51] K. Kawasaki and T. Fukaya , “Regulatory Landscape of Enhancer‐Mediated Transcriptional Activation,” Trends in Cell Biology 34 (2024): 826–837, 10.1016/j.tcb.2024.01.008. 38355349

[52] M. R. Mumbach , A. J. Rubin , R. A. Flynn , et al., “HiChIP: Efficient and Sensitive Analysis of Protein‐Directed Genome Architecture,” Nature Methods 13 (2016): 919–922, 10.1038/nmeth.3999. 27643841 PMC5501173

[53] F. Klein , N. Feldhahn , J. L. Mooster , et al., “Tracing the Pre‐B to Immature B Cell Transition in human Leukemia Cells Reveals a Coordinated Sequence of Primary and Secondary IGK Gene Rearrangement, IGK Deletion, and IGL Gene Rearrangement,” The Journal of Immunology 174 (2005): 367–375, 10.4049/jimmunol.174.1.367. 15611260

[54] S. Reckel , R. Hamelin , S. Georgeon , et al., “Differential Signaling Networks of Bcr–Abl p210 and p190 Kinases in Leukemia Cells Defined by Functional Proteomics,” Leukemia 31 (2017): 1502–1512, 10.1038/leu.2017.36. 28111465 PMC5508078

[55] J. A. Cutler , R. Tahir , S. K. Sreenivasamurthy , et al., “Differential Signaling through p190 and p210 BCR‐ABL Fusion Proteins Revealed by Interactome and Phosphoproteome Analysis,” Leukemia 31 (2017): 1513–1524, 10.1038/leu.2017.61. 28210003

[56] T. Kalkan , S. Bornelöv , C. Mulas , et al., “Complementary Activity of ETV5, RBPJ, and TCF3 Drives Formative Transition from Naive Pluripotency,” Cell Stem Cell 24 (2019): 785–801, 10.1016/j.stem.2019.03.017. 31031137 PMC6509416

[57] C. D. S. Katerndahl , L. M. Heltemes‐Harris , M. J. L. Willette , et al., “Antagonism of B Cell Enhancer Networks by STAT5 Drives Leukemia and Poor Patient Survival,” Nature Immunology 18 (2017): 694–704, 10.1038/ni.3716. 28369050 PMC5540372

[58] S. Kollmann , E. Grundschober , B. Maurer , et al., “Twins with Different Personalities: STAT5B—But Not STAT5A—Has a Key Role in BCR/ABL‐Induced Leukemia,” Leukemia 33 (2019): 1583–1597, 10.1038/s41375-018-0369-5. 30679796 PMC6755975

[59] L. N. Chan , M. A. Murakami , M. E. Robinson , et al., “Signalling Input from Divergent Pathways Subverts B Cell Transformation,” Nature 583 (2020): 845–851, 10.1038/s41586-020-2513-4. 32699415 PMC7394729

[60] J. C. Kim , M. Chan‐Seng‐Yue , S. Ge , et al., “Transcriptomic Classes of BCR‐ABL1 Lymphoblastic Leukemia,” Nature Genetics 55 (2023): 1186–1197, 10.1038/s41588-023-01429-4. 37337105 PMC10335939

[61] R. M. Meyers , J. G. Bryan , J. M. McFarland , et al., “Computational Correction of Copy Number Effect Improves Specificity of CRISPR–Cas9 Essentiality Screens in Cancer Cells,” Nature Genetics 49 (2017): 1779–1784, 10.1038/ng.3984. 29083409 PMC5709193

[62] L. Nicosia , G. J. Spencer , N. Brooks , et al., “Therapeutic Targeting of EP300/CBP by Bromodomain Inhibition in Hematologic Malignancies,” Cancer Cell 41 (2023): 2136–2153, 10.1016/j.ccell.2023.11.001. 37995682

[63] E. Searle , V. Campbell , C. Pawlyn , et al., “Tolerability and Clinical Activity of Novel First‐in‐Class Oral Agent, Inobrodib (CCS1477), in Combination with Pomalidomide and Dexamethasone in Relapsed/Refractory Multiple Myeloma,” Blood 144 (2024): 1023, 10.1182/blood-2024-199779.

[64] J. Ding , J. Romani , M. Zaborski , et al., “Inhibition of PI3K/mTOR Overcomes Nilotinib Resistance in BCR‐ABL1 Positive Leukemia Cells through Translational Down‐Regulation of MDM2,” PLoS ONE 8 (2013): 83510, 10.1371/journal.pone.0083510. PMC3859659 24349524

[65] H. Quentmeier , S. Eberth , J. Romani , M. Zaborski , and H. G. Drexler , “BCR‐ABL1‐Independent PI3Kinase Activation Causing Imatinib‐Resistance,” Journal of Hematology & Oncology 4 (2011): 6, 10.1186/1756-8722-4-6. 21299849 PMC3041785

[66] G. Corleone , C. Sorino , M. Caforio , et al., “Enhancer Engagement Sustains Oncogenic Transformation and Progression of B‐Cell Precursor Acute Lymphoblastic Leukemia,” Journal of Experimental & Clinical Cancer Research 43 (2024): 179, 10.1186/s13046-024-03075-y. 38926853 PMC11210131

[67] S. Narang , Y. Ghebrechristos , N. A. Evensen , et al., “Clonal Evolution of the 3D Chromatin Landscape in Patients with Relapsed Pediatric B‐Cell Acute Lymphoblastic Leukemia,” Nature Communications 15 (2024): 7425, 10.1038/s41467-024-51492-6. PMC11358475 39198446

[68] Y. Sun , J. Han , Z. Wang , X. Li , Y. Sun , and Z. Hu , “Safety and Efficacy of Bromodomain and Extra‐Terminal Inhibitors for the Treatment of Hematological Malignancies and Solid Tumors: a Systematic Study of Clinical Trials,” Frontiers in Pharmacology 11 (2020): 621093, 10.3389/fphar.2020.621093. 33574760 PMC7870522

[69] S. A. Abraham , L. E. M. Hopcroft , E. Carrick , et al., “Dual Targeting of p53 and c‐MYC Selectively Eliminates Leukaemic Stem Cells,” Nature 534 (2016): 341–346, 10.1038/nature18288. 27281222 PMC4913876

[70] J. Zhou , S. Wang , D. Nie , et al., “Super‐Enhancer Landscape Reveals Leukemia Stem Cell Reliance on X‐Box Binding Protein 1 as a Therapeutic Vulnerability,” Science Translational Medicine 13 (2021): abh3462, 10.1126/scitranslmed.abh3462. 34550724

[71] Z. Chen , S. Shojaee , M. Buchner , et al., “Signalling Thresholds and Negative B‐Cell Selection in Acute Lymphoblastic Leukaemia,” Nature 521 (2015): 357–361, 10.1038/nature14231. 25799995 PMC4441554

[72] I. M. Ward , K. Minn , J. van Deursen , and J. Chen , “p53 Binding Protein 53BP1 Is Required for DNA Damage Responses and Tumor Suppression in Mice,” Molecular and Cellular Biology 23 (2003): 2556–2563, 10.1128/MCB.23.7.2556-2563.2003. 12640136 PMC150747

[73] A. Kaneshige , L. Bai , M. Wang , et al., “A Selective Small‐Molecule STAT5 PROTAC Degrader Capable of Achieving Tumor Regression in Vivo,” Nature Chemical Biology 19 (2023): 703–711, 10.1038/s41589-022-01248-4. 36732620

[74] A. Kaneshige , Y. Yang , L. Bai , et al., “Discovery of AK‐1690: a Potent and Highly Selective STAT6 PROTAC Degrader,” Journal of Medicinal Chemistry 68 (2025): 5125–5151, 10.1021/acs.jmedchem.4c01009. 39311434

[75] R. Xu , H. Zhou , L. Bai , et al., “Discovery of SD‐436: a Potent, Highly Selective and Efficacious STAT3 PROTAC Degrader Capable of Achieving Complete and Long‐Lasting Tumor Regression,” Journal of Medicinal Chemistry 67 (2024): 20495–20513, 10.1021/acs.jmedchem.4c01946. 39509603 PMC12981319

[76] E. Buehler , Y. C. Chen , and S. Martin , “C911: A Bench‐Level Control for Sequence Specific siRNA Off‐Target Effects,” PLoS ONE 7 (2012): 51942, 10.1371/journal.pone.0051942. PMC3522603 23251657

[77] M. Pfeifer , R. Brem , T. P. Lippert , et al., “SSB1/SSB2 Proteins Safeguard B Cell Development by Protecting the Genomes of B Cell Precursors,” The Journal of Immunology 202 (2019): 3423–3433, 10.4049/jimmunol.1801618. 31085591 PMC6545462

[78] H. L. Ng , M. E. Robinson , P. C. May , A. J. Innes , C. Hiemeyer , and N. Feldhahn , “Promoter‐Centred Chromatin Interactions Associated with EVI1 Expression in EVI1 +3q− Myeloid Leukaemia Cells,” British Journal of Haematology 204 (2024): 945–958, 10.1111/bjh.19322. 38296260

[79] P. Freire‐Pritchett , H. Ray‐Jones , M. Della Rosa , et al., “Detecting Chromosomal Interactions in Capture Hi‐C Data with CHiCAGO and Companion Tools,” Nature Protocols 16 (2021): 4144–4176, 10.1038/s41596-021-00567-5. 34373652 PMC7612634

[80] S. A. Lambert , A. Jolma , L. F. Campitelli , et al., “The Human Transcription Factors,” Cell 175 (2018): 598–599, 10.1016/j.cell.2018.09.045. 30290144

[81] A. Bernardini , M. Lorenzo , M. Nardini , R. Mantovani , and N. Gnesutta , “The Phosphorylatable Ser320 of NF‐YA Is Involved in DNA Binding of the NF‐Y Trimer,” The FASEB Journal 33 (2019): 4790–4801, 10.1096/fj.201801989R. 30589568

