# Supplementary material for: BCR::ABL1‐Induced Enhancer Reprogramming Uncovers Hypersensitivity of Ph+B‐ALL Cells to Enhancer‐Targeting Drugs
Source: Adv Sci (Weinh). 2026 Mar 1;13(25):e17231. doi: 10.1002/advs.202517231 (PMC13137836; doi:10.1002/advs.202517231)
Supplement: Supplementary file 1 — Supporting File 1: advs74523‐sup‐0001‐SuppMat.pdf. [file ADVS-13-e17231-s001.pdf]

Supplementary Figures

BCR::ABL1-INDUCED ENHANCER REPROGRAMMING UNCOVERS HYPERSENSITIVITY OF PH+B-ALL CELLS TO ENHANCER-TARGETING DRUGS

Han Leng Ng[1,#], Trudy L. Glaser[1,#], Jintao Zhu[1], Mark E. Robinson[2], Kadriye N. Cosgun[2], Valeriya Malysheva[3,4,5], Ozgen Deniz[6], Nicholas T. Crump[1,7], Kaiyue Helian[1], Andrew J. Innes[1], Richard Burt[1], Li Sun[1], George John[1], Haibin Zhou[8], Atsunori Kaneshige[8], Longchuan Bai[8], Shaomeng Wang[8], Mikhail Spivakov[3,4], Markus Mueschen[2] and Niklas Feldhahn[1,*].

Affiliations:

[1]Centre for Haematology, Department of Immunology and Inflammation, Faculty of Medicine, Imperial College London, London, UK
[2]Center of Molecular and Cellular Oncology, Yale University, New Haven, USA
[3]MRC London Institute of Medical Sciences, London, UK
[4]Institute of Clinical Sciences, Faculty of Medicine, Imperial College, London, UK
[5]Present address: VIB Center for Molecular Neurology, University of Antwerp, Belgium and VIB Center for AI and Computational Biology, Leuven, Belgium
[6]Barts Cancer Institute, Queen Mary University of London, Charterhouse Square, London, UK
[7]The Hugh and Josseline Langmuir Centre for Myeloma Research, Imperial College London, London, UK
[8]Department of Internal Medicine, Medical School, University of Michigan, Ann Arbor, Michigan, USA

# Equal contribution
* Correspondence: n.feldhahn@imperial.ac.uk

This section includes 6 Supplementary Figures.

A separate document contains Supplementary Tables S1-18.

Supplementary Figure 1 (related to Figure 1):

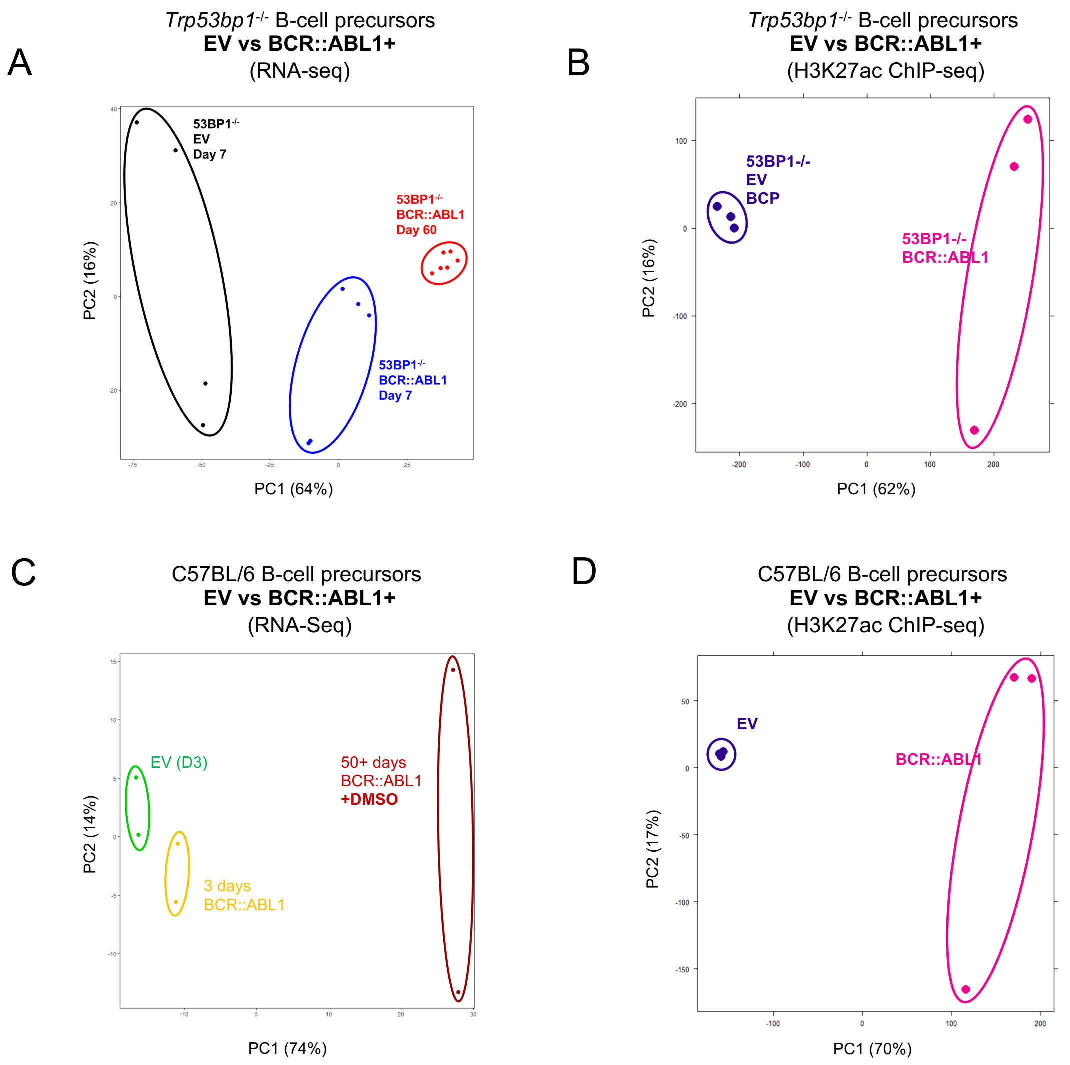

Supplementary Figure 1: *Transcriptional and enhancer reprogramming during BCR::ABL1-induced malignant transformation of murine B-cell precursors.* XY plots of Principal Component Analyses (PCA) for RNA-Seq (A/C) and H3K27ac ChIP-Seq (B/D) experiments are shown. Analyses were done on B-cell precursors (BCPs) from *Trp53bp1*$^{-/-}$ mice (A/B) or C57BL/6 wild type mice (C/D) that were either transduced with empty MIGR1 vectors (EV) or BCR::ABL1-encoding MIGRI vectors. Cells represent different experimental time points as outlined in Figure 1, with EV and early transformed cells collected either at 7 days post BCR::ABL1 transduction (for *Trp53bp1*$^{-/-}$ BCPs) or 3 days post transduction (for C57BL/6 BCPs), and fully transformed cells collected at ~60 days post transduction.

Supplementary Figure 2 (related to Figure 2):

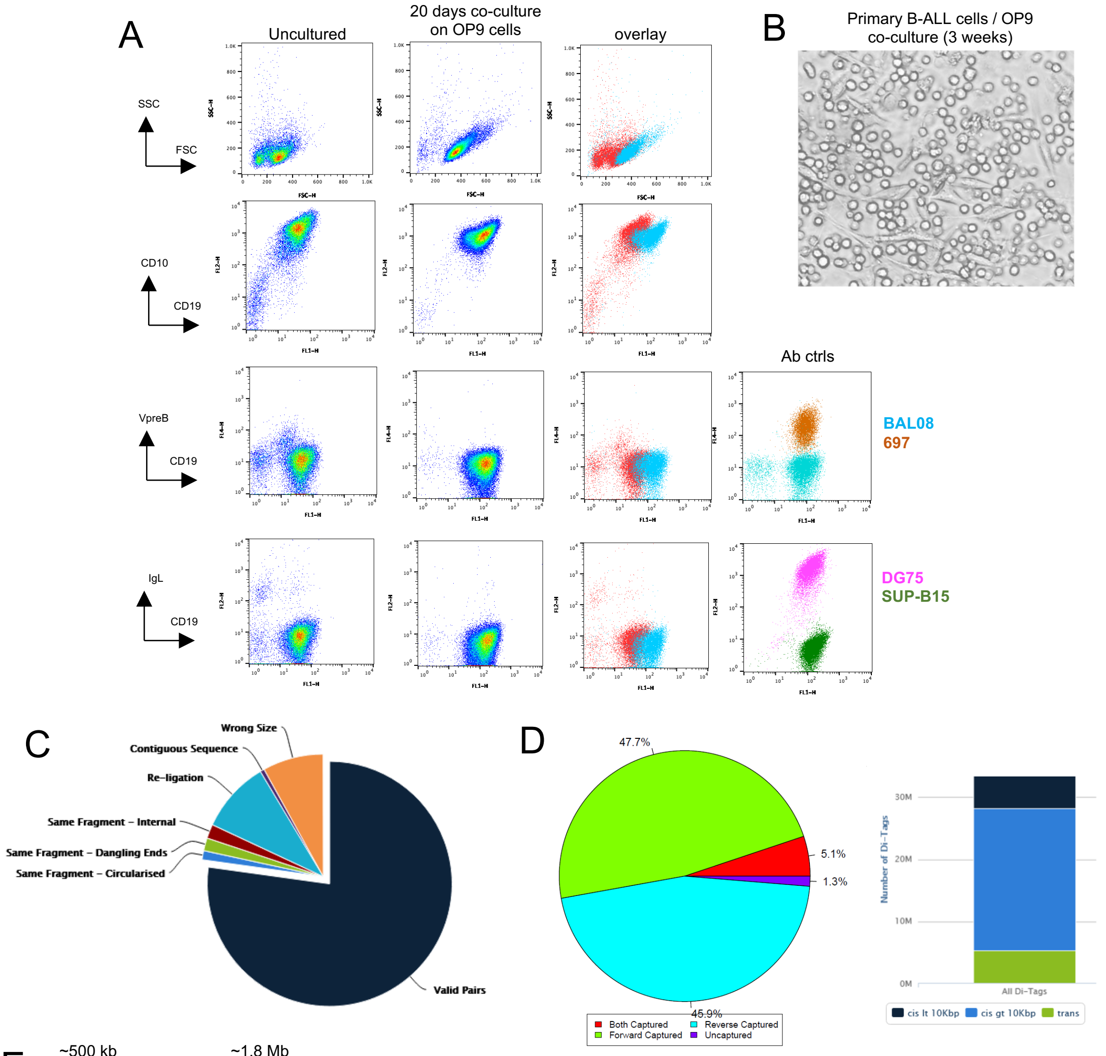

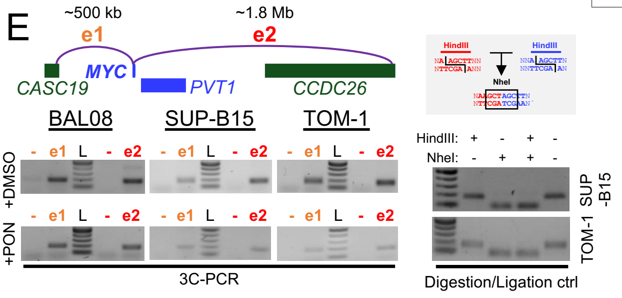

F

Total Reads	Pair reads	Valid pairs	Unique di-tag	% captured reads		
52,561,988	43,202,417	33,355,746	26,253,108	98.7%	+DMSO	SUP-B15
65,138,622	53,703,619	42,746,086	29,570,479	98.48%	+PON	
68,927,830	57,002,345	43,710,624	29,660,677	98.51%	+DMSO	TOM-1
76,008,211	61,144,156	40,564,123	25,226,269	97.67%	+PON	
65,271,817	54,989,654	40,788,584	27,393,198	98.36%	+DMSO	BAL08
68,593,925	57,777,489	44,245,792	30,292,832	98.83%	+PON	

Supplementary Figure 2: ***Analysis of enhancer-promoter interactions by PCHi-C and associated A-485 treatments.*** (A) Flow cytometry plots showing the preservation of primary Ph+B-ALL cells (BAL08) during 20 days of ex vivo co-culture on sub-lethally irradiated OP9 cells. Cells were stained with antibodies as indicated. First column shows uncultured BAL08 cells, second column shows BAL08 after 20 days of co-culture, third column shows an overlay of first and second columns (red = uncultured, turquoise = 20 days cultured). Cultured BAL08 cells are slightly larger in size (by FSC) and express slightly higher levels of CD19 compared to uncultured cells. The fourth column shows antibody positive control staining for the VpreB and IgL antibodies. Pre-B cell receptor-positive 697 Ph-negative B-ALL cells (brown) were used as positive control for VpreB, DG75 Burkitt's lymphoma cells (pink) were used as positive control for IgL. Positive controls were shown as overlays with cells that are negative for the respective antibodies in the colors indicated. (B) A representative image of primary Ph+B-ALL cells (BAL08) ex vivo co-cultured for 20 days on sub-lethally irradiated OP9 cells is shown. (C-F) Different parts of the quality control of PCHi-C samples are shown, with (C) indicating the quality of the Hi-C procedure, (D) indicating the quality of the promoter-capture step, (E) showing representative agarose gel images that validate long-range chromatin interaction capture (left; 3C-PCR for *MYC*-to-enhancer interactions as examples) and 3C digestion/ligation steps (right), and (F) indicating the total numbers of reads sequenced and processed. Note that the proportion of captured di-tags was ~98.7% (F), of which an average of 84.3% were in cis interactions.

Supplementary Figure 2 (continued):

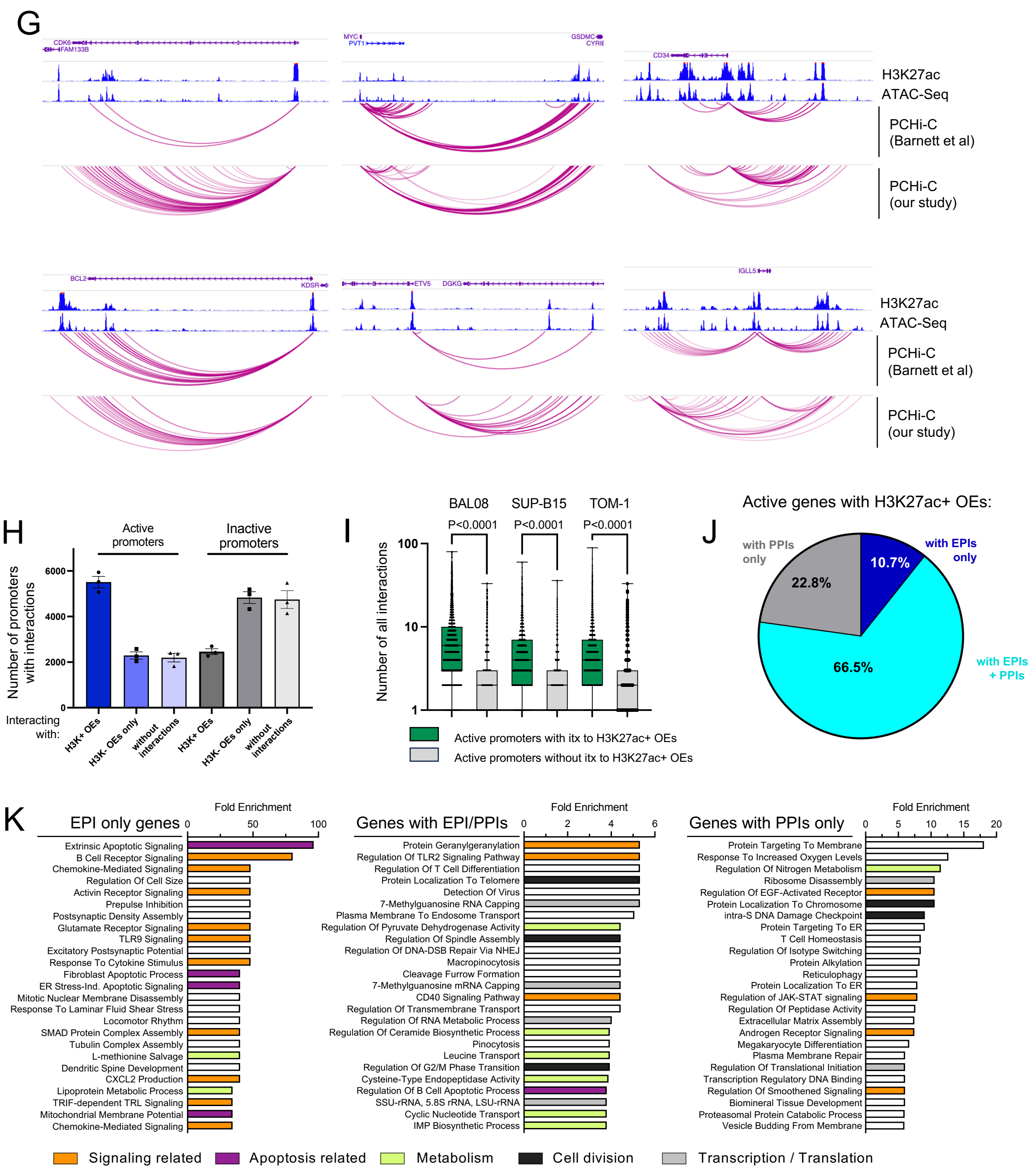

Supplementary Figure 2 (continued): (G) A comparison of PCHi-C interactions obtained by our study and by Barnett et al for the Ph+B-ALL cell line SUP-B15 is shown for 6 different loci. (H) Bar chart showing the number of active versus inactive promoters with PCHi-C interactions that do or do not display interactions with at least one H3K27ac+ OE (i.e., H3K+ OE vs H3K- OE) and promoters without PCHi-C-defined interactions. Individual dots represent numbers obtained for the three samples analyzed by PCHi-C (i.e., BAL08, SUP-B15, TOM-1) and the mean ± SEM is shown. (I) Box-and-whisker plot showing the total numbers of interactions for active promoters that do or do not display interactions (itx) with at least one H3K27ac+ OE (i.e., H3K+ OE vs H3K- OE). Number of interactions include promoter interactions without H3K27ac+ OEs. Statistical analysis was performed using GraphPad PRISM unpaired Student's t-test. Box represents median and interquartile range (25th – 75th percentile), with the whiskers showing the minimum and maximum. (J) A pie chart is shown indicating the average % of active genes with H3K27ac+ OEs in Ph+B-ALL cells with EPIs only, EPIs+PPIs, or PPIs only. (K) The 25 most enriched Gene Ontology (GO) pathways (biological processes) for active genes with PCHi-C interactions are shown. Pathway associations to functional groups are indicated by color. Pathway names are shortened to match the figure width.

Supplementary Figure 2 (continued):

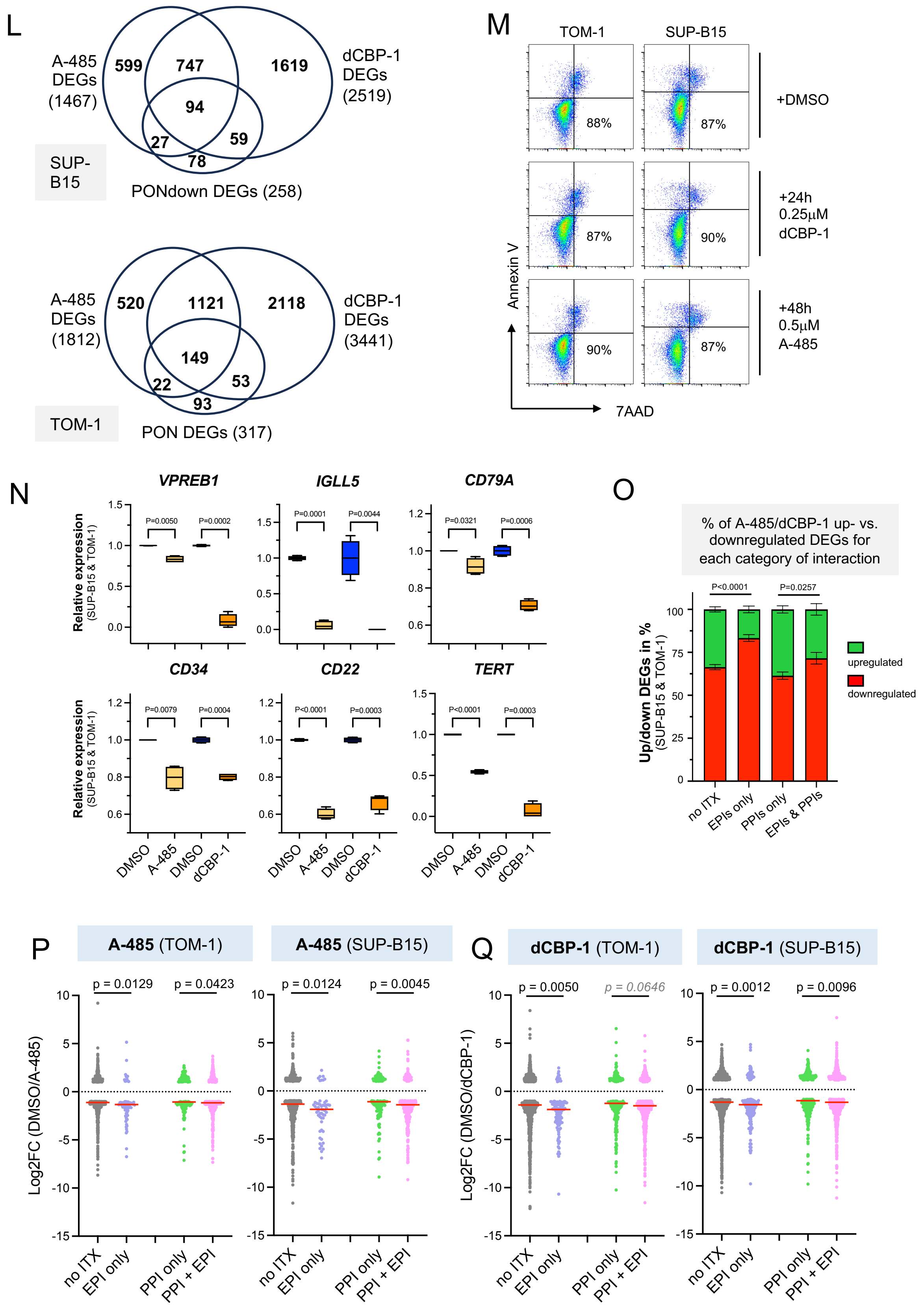

Supplementary Figure 2 (continued): (L) A diagram is shown indicating the overlap of dCBP-1 DEGs, A-485 DEGs and Ponatinib (PON) DEGs for the experiments performed in TOM-1 and SUP-B15 cells. (M) CBP/P300 was inhibited in Ph+B-ALL cells with sub-lethal concentrations/times of A-485 (0.5 μM for 48 h) and dCBP-1 (0.25 μM for 24 h). Representative flow cytometry plots for the treatments of are shown, with Annexin V and 7AAD indicating apoptotic and dead cells, respectively. (N) Bar diagrams showing relative expression values from A-485 or dCBP-1 treated compared to DMSO treated TOM-1 and SUP-B15 cells defined by RNA-Seq (DMSO and 0.5 μM A-485 for 48 h, or DMSO and 0.25 μM dCBP-1 for 24 h). To allow joint comparison, values were normalized to average DMSO. Indicated genes are lineage-, cell type- or differentiation stage-specific genes. (O) Percentages of A-485 and dCBP-1 up- and downregulated DEGs by RNA-Seq with respect to all DEGs are shown. Data represents values of two treatments (A-485 and dCBP-1) performed for TOM-1 and SUP-B15, respectively, with n=2 for each condition. (P/Q) Log2 fold change (Log2FC) values of A-485/DMSO DEGs (0.5 μM for 48 h) or dCBP-1/DMSO DEGs (0.25 μM for 24 h) from TOM-1 and SUP-B15 cells (n=2) are plotted for genes with neither EPIs nor PPIs (no ITX), genes with EPIs in comparison to genes with PPIs, and genes with EPIs+PPIs. The red line represents the mean. Statistical analysis was performed by paired Student's t-test and GraphPad PRISM.

Supplementary Figure 3 (related to Figure 3):

A

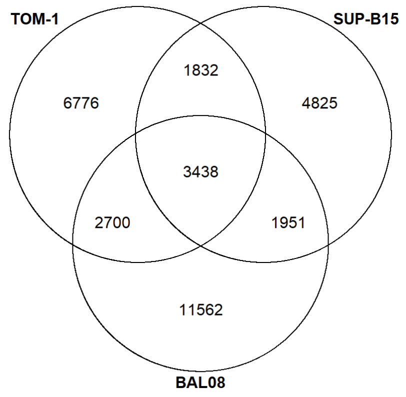

Ph+B-ALL CORE interactions by PCHi-C and H3K27ac-ChIP-Seq:

B

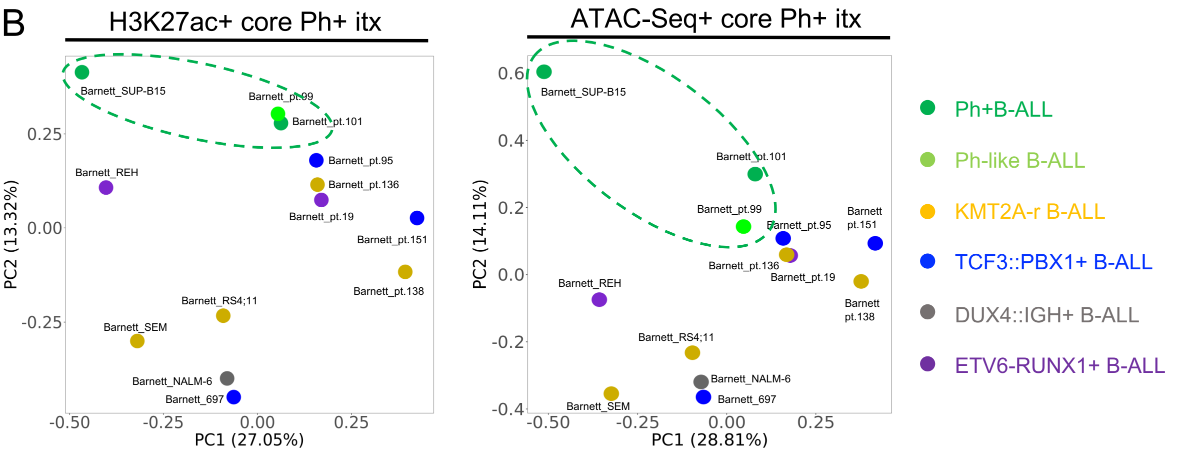

Supplementary Figure 3: *'Ph+B-ALL-defining core interactions' by PCHi-C and H3K27ac ChIP-seq*. (A) A Venn diagram is shown indicating the overlap of enhancer-promoter interactions defined by PCHi-C and H3K27ac ChIP-seq and detected in three Ph+B-ALL specimen as indicated. The center indicates interactions shared by all samples that were considered as Ph+B-ALL core interactions. (B) Two PCA plots are shown that use PCHi-C data from *Barnett et al* and 'Ph+B-ALL CORE interactions' to allow separation of Ph+ (and Ph-like) from Ph-negative B-ALL cells. Analysis focusses on the main B-ALL subtypes as indicated. PCHi-C data from cell lines and cells from primary B-ALL patients was filtered for PCHi-C interactions present in all Ph+B-ALL samples of the dataset and overlapped with H3K27ac (left) of ATAC-Seq peaks (right) at both ends of the PCHi-C interaction.

Supplementary Figure 4 (related to Figure 4):

A

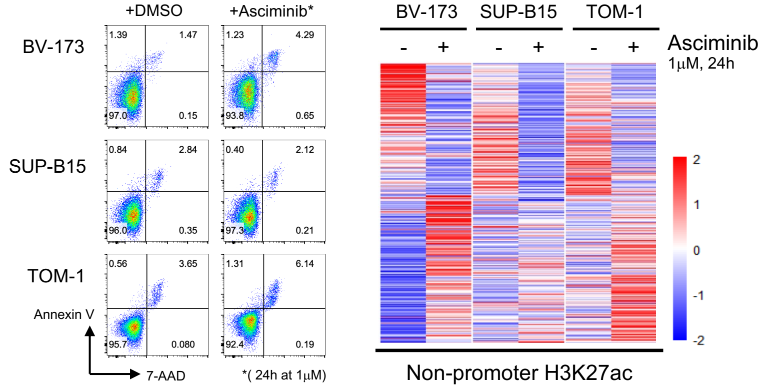

B

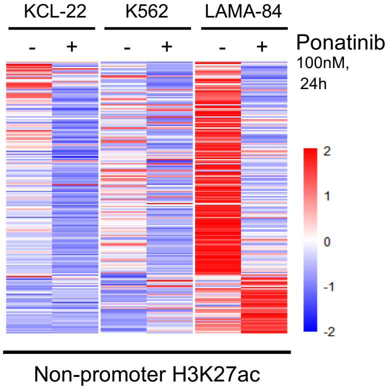

Supplementary Figure 4: ***24h BCR::ABL1 kinase inhibition causes changes in non-promoter H3K27ac signals in Ph+B-ALL and CML cell lines .*** (A) (Left) Flow cytometry images of Annexin V and 7-AAD stained cells are shown for three Ph+B-ALL cell lines treated for 24 h with 1 μM Asciminib or DMSO, documenting that this treatment does not substantially alter the viability during the 24 h period. (Right) A Heatmap of H3K27ac ChIP-Seq signals at non-promoter regions is shown for the cells and treatment on the left. Only regions with differential H3K27ac signals in all samples are plotted. (B) A heatmap of H3K27ac ChIP-Seq signals at non-promoter regions as in (A) is shown, but for three CML cell lines and using Ponatinib as in Figure 4B/C.

Supplementary Figure 5 (related to Figure 5):

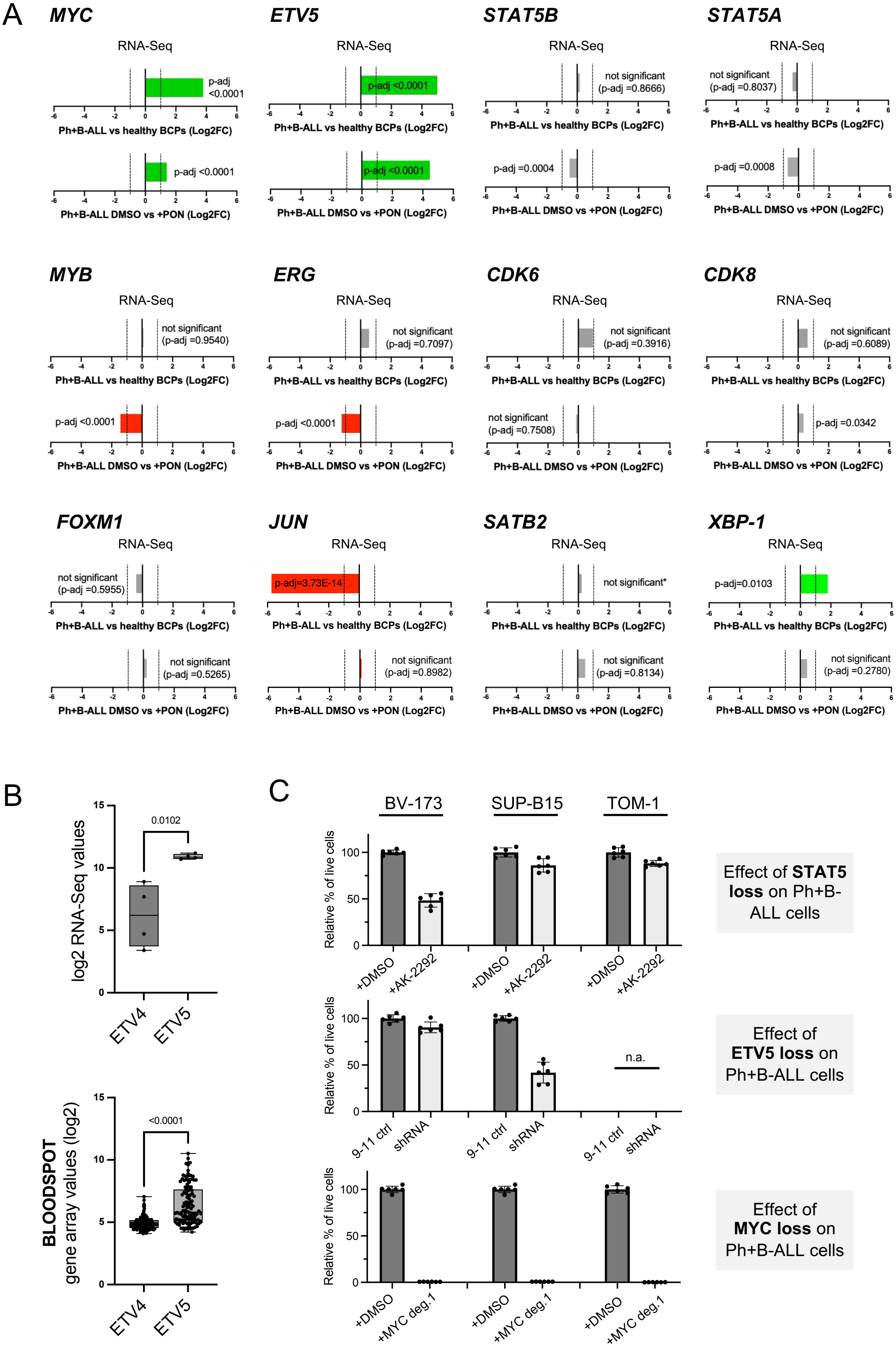

Supplementary Figure 5 (continued):

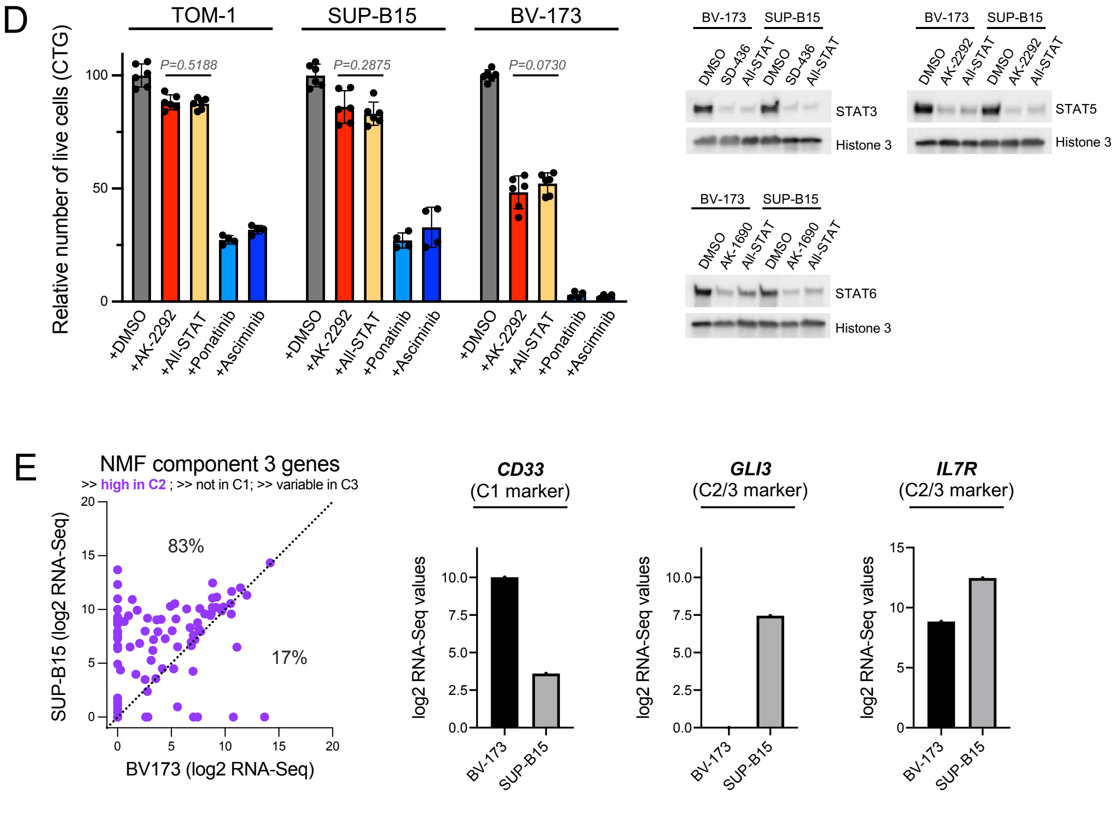

Supplementary Figure 5: ***Assessment of transcription factor (TF) expression and dependencies in Ph+B-ALL cells.*** (A) Bar charts showing the data from Figure 5A but separately for individual TFs previously linked to Ph+B-ALL as indicated. Top bars show Log2FC values for comparison of Ph+B-ALL cells to healthy bone marrow BCPs [HBM]. Bottom bars show Log2FC values for comparison of DMSO treated vs Ponatinib (PON) treated Ph+B-ALL cells. For DMSO/PON, three Ph+B-ALL cell lines (BV-173, TOM-1, SUP-B15) and one patient cells from one Ph+B-ALL patient (BAL08) were compared. For Ph+B-ALL/HBM, the same Ph+B-ALL cells were compared to CD19+CD10+ HBMs isolated from n=3 healthy donors. (B) (Top) A bar diagram visualizes RNA-Seq expression values for ETV4 and ETV5 from Ph+B-ALL cell lines and primary cells used in this study (TOM-1, SUP-B15, BV-173 and BAL08). (Bottom) ETV4 and ETV5 expression values of primary Ph+B-ALL cells from the Microarray Innovations in Leukemia (MILE) Study obtained through the BLOODSPOT database (https://www.fobinf.com/) are shown. (C) Bar diagrams are shown visualizing relative numbers of live cells obtained from n≥2 4-day Cell Titer Glo (CTG) experiments of Ph+B-ALL cell lines treated with the STAT5 degrader AK-2292 (2 µM; top panel), experiencing ETV5 silencing via RNAi (middle panel), or treated with the MYC degrader A80.2HCl/MYC degrader 1 (25 nM; bottom panel). For RNAi experiments, a C911 seed control was used, which is the same shRNA as the ETV5 targeting shRNA but mutated at nucleotide positions 9-11. C911 seed control shRNAs are predicted to lack on-targeting activity but have the same off-target activity. Note, that RNAi could not be applied to TOM-1 cells due to their immediate, anti-proliferative response to any viral transduction. Statistical analysis was performed using Student's t-test and GraphPad PRISM, with mean ± SD shown. (D) (left) A bar diagram is shown visualizing relative numbers of live cells obtained from 4-day Cell Titer Glo (CTG) experiments comparing STAT5 degradation to combined degradation of STAT5, STAT3 and STAT6. The diagram summarizes the results of n≥2 experiments with n=2 technical replicates each for TOM-1, SUP-B15 and BV-173 Ph+B-ALL cells treated with the STAT5 degrader AK-2292 (2 µM), a mix of AK-2292 (2 µM), the STAT3 degrader SD-436 (0.4 µM) and the STAT6 degrader AK-1690 (1.5 µM) [All-STAT], the TKIs Ponatinib (100 nM) or Asciminib (100 nM), or DMSO as control. Drugs were replenished with a 1:1 media change on D2 for all CTG experiments in this study. Statistical analysis was performed using paired Student's t-test and GraphPad PRISM, with mean ± SD shown. (Right) Western blot validation of the degraders used by CTG, in combination or individually, performed on BV-173 and SUP-B15 cells. (E) (Left) XY plot showing log2-transformed normalized RNA-Seq expression values from BV-173 and SUP-B15 cells for NMF component 3 genes defined by Kim et al, which are highly expressed by the Ph+B-ALL C2 subgroup, not or little expressed by Ph+B-ALL C1 subgroup and variable expressed by Ph+B-ALL C3 subgroup. (Right) Bar charts of genes described by Kim et al as expressed in the Ph+B-ALL C1-C3 subgroups as indicated.

Supplementary Figure 6 (related to Figure 6):

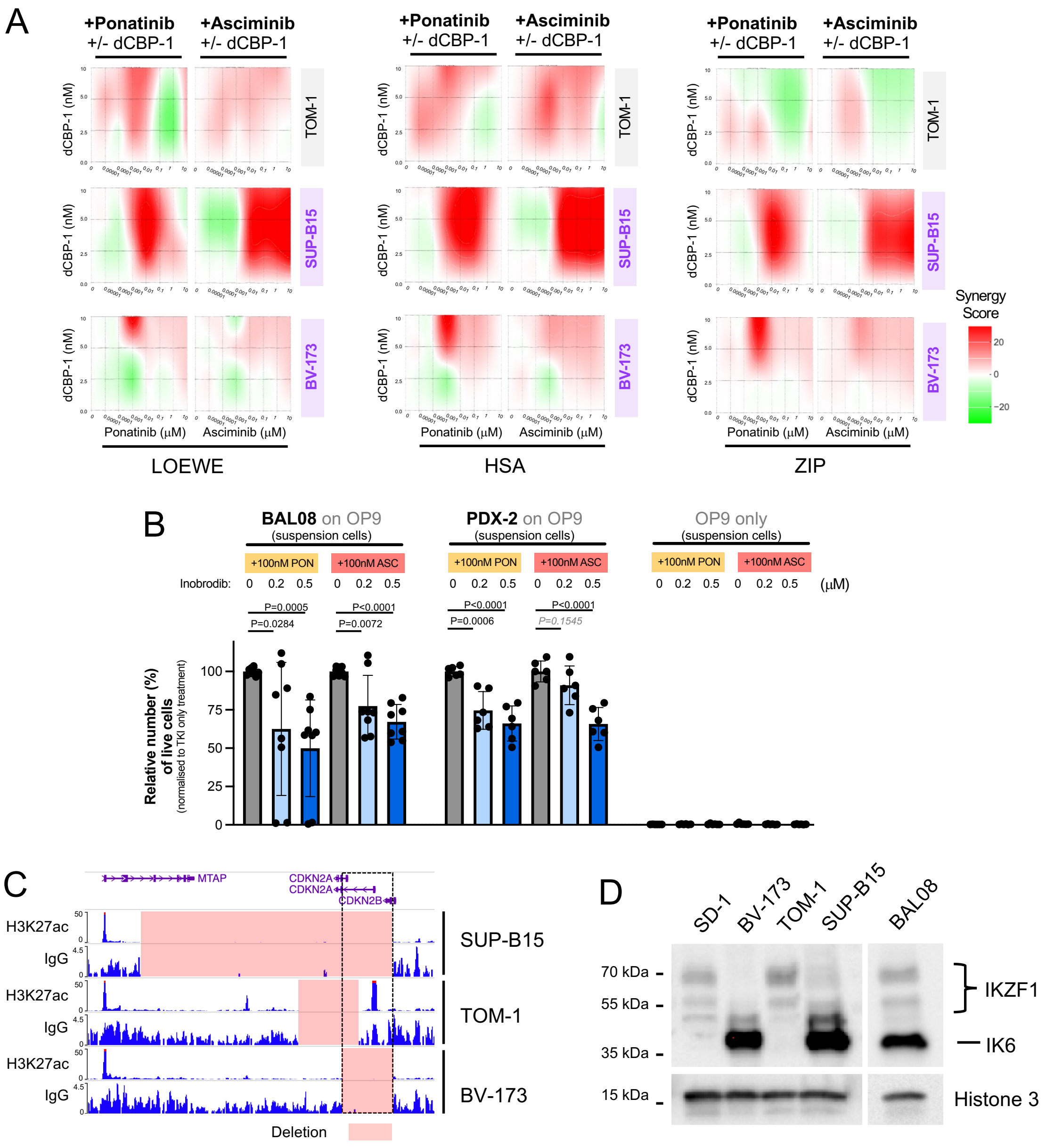

E

Ph+B-ALL	*CDKN2A/B*	*IKZF1*	*PAX5*	*IKZF1*[Plus]
SD-1	germline	germline*	germline	no
BV-173	deleted	IK6*	germline	yes
TOM-1	deleted	germline	germline	no
SUP-B15	deleted	IK6	germline	yes
BAL08	germline	IK6	germline	no

*Iacobucci et al 2008, https://doi.org/10.1182/blood-2007-09-112631

Supplementary Figure 6: Combined treatment of TKIs +/- dCBP-1/Inobrodib and *IKZF1*[PLUS] *classification on Ph+B-ALL lines and patients used in this study.* (A) A synergy analysis for the combination treatment of Ponatinib or Asciminib with different concentrations of dCBP-1 as indicated is shown. XY plots show LOEWE, HSA and ZIP scores generated by SynergyFinder using the data from Figure 6F. BLISS scores can be found in Figure 6G. (B) A Bar diagram is shown indicating the relative number of live cells determined by Cell Titer Glo (CTG) assay for primary Ph+B-ALL cells cultured for four days on mitotically inactivated OP-9 cells in the presence of the drugs indicated (n=6-8, i.e., 3-4 individual treatments with 2 technical controls each). CTG values were normalized to 'TKI only' treated cells (PON or ASC) to indicate the effect of Inobrodib addition to TKI treatment. Statistical analysis was performed using unpaired student's t-test and GraphPad Prism, with mean ± SD shown. (C) ChIP-Seq custom tracks for anti-H3K27ac and IgG control antibodies are shown for SUP-B15, TOM-1 and BV-173 cells depicting the *CDKN2A/B* locus. Pink areas show nearly complete absence of any background read alignment indicative of homozygous genomic deletion. (D) Western blot of IKZF1 and Histone 3 as loading control indicating cell lines/patients expressing full-length IKZF1 and/or the dominant-negative isoform IK6. (E) Summary of *CDKN2A/B* and *IKZF1* status defining cells as *IKZF1*[PLUS] or not.
